# Comprehensive Evaluation of Differential Methylation Analysis Methods for Bisulfite Sequencing Data

**DOI:** 10.3390/ijerph18157975

**Published:** 2021-07-28

**Authors:** Yongjun Piao, Wanxue Xu, Kwang Ho Park, Keun Ho Ryu, Rong Xiang

**Affiliations:** 1 School of Medicine, Nankai University, Tianjin 300071, China; ypiao@nankai.edu.cn; 2 Tianjin Key Laboratory of Human Development and Reproductive Regulation, Tianjin Central Hospital of Gynecology Obstetrics, Tianjin 300199, China; 3 Center for Reproductive Medicine, Department of Obstetrics and Gynecology, Peking University Third Hospital, Beijing 100191, China; xwx@mail.nankai.edu.cn; 4 Department of Computer Science, College of Electrical and Computer Engineering, Chungbuk National University, Cheongju 28644, Korea; khblack@dblab.chungbuk.ac.kr; 5 Faculty of Information Technology, Ton Duc Thang University, Ho Chi Minh City 700000, Vietnam

**Keywords:** differentially methylated regions, DNA methylation, BS-seq

## Abstract

**Background:** With advances in next-generation sequencing technologies, the bisulfite conversion of genomic DNA followed by sequencing has become the predominant technique for quantifying genome-wide DNA methylation at single-base resolution. A large number of computational approaches are available in literature for identifying differentially methylated regions in bisulfite sequencing data, and more are being developed continuously. **Results:** Here, we focused on a comprehensive evaluation of commonly used differential methylation analysis methods and describe the potential strengths and limitations of each method. We found that there are large differences among methods, and no single method consistently ranked first in all benchmarking. Moreover, smoothing seemed not to improve the performance greatly, and a small number of replicates created more difficulties in the computational analysis of BS-seq data than low sequencing depth. **Conclusions:** Data analysis and interpretation should be performed with great care, especially when the number of replicates or sequencing depth is limited.

## 1. Background

DNA methylation is a major epigenetic marker that involves the transfer of a methyl group to the C5 carbon residue (5 mC) of cytosines, mediated by a family of DNA methyltransferases [1]. A number of cytosine variants, such as 5-hydroxymethylcytosine [2,3], 5-formylcytosine, and 5-carboxylcytosine [4,5], have also been discovered. DNA methylation plays an important role in various biological processes [6], e.g., the regulation of gene expression [7,8], genomic imprinting [9,10], cell differentiation [11,12], development [13,14], and inflammation [15]. Aberrant methylation has been reported to be associated with various diseases and cancer [16]. Understanding the functional role of DNA methylation is therefore of great importance. As next-generation sequencing technologies have advanced, the bisulfite conversion of genomic DNA followed by sequencing (BS-seq) has become the predominant technique for quantifying genome-wide DNA methylation at single-base resolution. The treatment of DNA with sodium bisulfite converts unmethylated cytosines (Cs) into uracils (Us) while keeping methylated cytosines unchanged, and the uracils are read as thymines (Ts) by high-throughput sequencing. The millions of reads produced by the sequencer are then mapped back to a reference genome with bisulfite read aligners, such as Bismark [17], BSMAP [18], and BatMeth [19]. Various studies have previously addressed the detailed issues in mapping algorithms and compared their performance [20,21]; these topics are not addressed further here. After mapping, the methylation of each CpG site can be quantified by summarizing the frequency of Cs in the total number of reads (Cs + Ts) mapped to that locus.

In general, the fundamental use of BS-seq is in the identification of differentially methylated regions (DMRs), that is, genomic regions that show significant differences in methylation levels between distinct biological or medical conditions, e.g., normal vs. disease. The differential methylation analysis of BS-seq data generally consists of two steps: the identification of differentially methylated cytosines (DMCs) using a statistical test and the grouping of cytosines into regions with a specific segmentation method. Note that the above two steps can be carried out regardless of the order; the statistical testing can be conducted after merging nearby GpG sites into regions. Accurately identifying DMRs from BS-seq data is a nontrivial task, and it presents several challenges due to (i) limitations of the number of replicates and sequencing depth, (ii) both technical and biological variations, and (iii) the large volume of whole-genome BS-seq data, which is computationally expensive. To address the above issues, several computational approaches have been developed for DMR detection, including Fisher’s exact test [22], BSmooth [23], methylKit [24], methylSig [25], DSS [26], metilene [27], RADMeth [28], and Biseq [29]. However, it is difficult to choose an appropriate analysis method due to the lack of benchmarking. The computational approaches for DMR detection in BS-seq have been much less comprehensively evaluated than those in other sequencing applications, such as peak calling in ChIP-seq [30,31,32] and differentially expressed gene detection in RNA-seq [33,34,35,36,37]. The effect of key features in next-generation sequencing experiments, such as library size and the number of replicates, on BS-seq data analysis remains to be tested.

The tools for benchmarking include Fisher’s exact test [22], BSmooth [23], methylKit [24], methylSig [25], DSS [26], metilene [27], RADMeth [28], and Biseq [29]. Note that RRBS-Analyser [38], Methy-Pipe [39], and Bisulfighter [40] have been excluded in our analysis, since the download link of these tools provided in their manuscript did not work. All methods take methylation quantification data as an input that contains the number of methylated and unmethylated reads mapped to each CpG dinucleotide in each replicate. Fisher’s exact test, BSmooth, DSS, RADMeth, and Biseq work directly on methylation count, while methylKit, methylSig, and metilene need to transform the methylation count to a percentage. A brief summary of the tools used for benchmarking is presented in Table 1. Fisher’s exact test [22] is the first approach used for differential methylation analysis. Unlike the other methods, Fisher’s exact test is implemented in conjunction with other software packages, e.g., methylKit and BSmooth, instead of being developed as an independent one. The resulting *p*-value is directly used as the DMC cutoff criterion. BSmooth [23] employs a local likelihood smoothing strategy to estimate methylation profiles based on the assumption that the methylation levels of neighboring CpGs change smoothly. The method uses the smoothed methylation profiles to compute the *t*-like statistic of each CpG site and combines consecutive CpGs by a cutoff to form DMRs. MethylKit [24] models the methylation level of a CpG or a region using logistic regression and tests the difference in log odds between the treatment and control groups to determine DMCs/DMRs. A sliding window-based segmentation method is implemented in methylKit to merge neighboring CpGs with a predefined window size. In addition to differential analysis, the package also provides several useful functions, such as the hierarchical clustering of samples, principal component analysis, and annotation of DMRs. MethylSig [25] determines differential methylation using likelihood ratio estimation based on a beta-binomial model and provides the option of using information from nearby CpGs to improve the model parameter estimation. The method also uses the sliding window approach to segment the genome into subregions for DMR analysis. DSS [26] describes the BS-seq count by a Bayesian hierarchical model based on the beta-binomial distribution, and the Wald test is adopted to test the hypothesis of each CpG dinucleotide. DMRs are then defined based on several parameters, such as minimum length, minimum number of CpGs, and minimum number of significant CpGs. Metilene [27] is a nonparametric method that does not make any assumptions about the data distribution. The method iteratively segments the genome based on a circular binary segmentation algorithm, calculates the *p*-values of each segmented window using a two-dimensional Kolmogorov–Smirnov test, and uses the resulting *p*-values as the iteration end condition. RADMeth [28] models the methylation level of each site using beta-binomial regression and uses the maximum likelihood method to estimate the distribution parameters. The significance of differential methylation is assessed by the log-likelihood ratio test, and the *p*-values are then transformed using the weighted Z test. The correlation coefficients of the *p*-values are calculated to combine significant DMCs within the predefined window. Biseq [29] works by identifying CpG clusters as sets of consecutive CpGs satisfy several predefined conditions. The methylation levels of the CpGs within a CpG cluster are then smoothed based on the weighted local likelihood approach, and the Wald test based on the beta regression model is used to determine the significance of the differential methylation at each CpG site. More detailed descriptions of the tools and their statistical models may be obtained by referring to the original publications. The analysis procedure of each method and the parameter usage followed the recommendations provided in the tool manual or default settings.

In this article, we have focused on a comprehensive evaluation of eight commonly used differential methylation analysis methods and have described the potential advantages and drawbacks of each method. We first assessed the performance in terms of the true positive rate and examined how variations in sequencing depth and the number of replicates influence the interpretation of BS-seq experiments. We also evaluated the false positives of each method when applied to simulated datasets containing no DMRs. Moreover, two additional benchmark datasets from the mouse and human methylome were used to test the DMR detection power and boundary estimation ability on real biological data. An integrated analysis of BS-seq, RNA-seq, and DNase-seq was also conducted between IMR90 human lung fibroblasts (IMR90) and H1 human embryonic stem cells (H1-hESCs). The results demonstrated large differences among methods in the detection of DMCs/DMRs in both simulated and real datasets. No single method consistently ranked first in all benchmarking. Moreover, smoothing did not greatly improve the performance, and a small number of replicates introduced more difficulties in computational analysis of BS-seq data than did a low sequencing depth. Data analysis and interpretation should be performed with great care, especially when the number of replicates or the sequencing depth is limited.

## 2. Results and Discussion

### 2.1. Assessment of Performance in Detecting Differentially Methylated Cytosines

**Figure 1:**
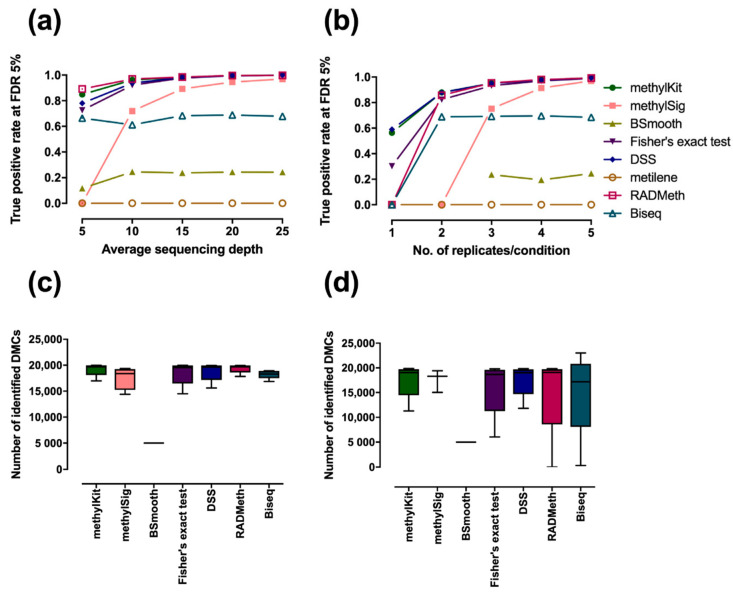
Comparison of methods for individual DMC detection. The true positive rate was reported at a 5% Benjamini–Hochberg adjusted *p*-value on different simulated data for variation in (**a**) the average sequencing depth: 5×, 10×, 15×, 20×, and 25× (three replicates/conditions in all cases) and (**b**) the number of replicates per condition: 1, 2, 3, 4, and 5 (10× coverage in all cases). The number of DMCs detected by each method in the data with 5 different sequencing depths and 5 different numbers of replicates are summarized in (**c**,**d**), respectively. Note that some datapoints are absent in (**b**) since these tools have minimum replicate requirements, i.e., Bsmooth requires at least 3 and methylSig at least 2 replicates in each condition. The total number of CpGs in each simulated dataset was 100K, and the number of DMCs was set to 20%. The methylation difference in DMCs between two conditions was randomly selected from a range of weak to strong signals (0.2 to 1). DMCs were directly extracted by each method with its default parameters without applying any segmentation or clustering strategies for merging CpGs into regions. The Benjamini–Hochberg [42] procedure was used to adjust *p*-values for all methods to correct for multiple testing bias.

DMCs were directly extracted on cytosine-based simulated data by each method with its default parameters without applying any segmentation or clustering strategies for merging CpGs into regions, and the true positive rate of each method at a Benjamini–Hochberg adjusted *p*-value of 5% was determined, with variation in the average sequencing depth (Figure 1a) and the number of replicates (Figure 1b). True positives (TP) were defined as correct identification of DMCs, false negatives (FN) were defined as the incorrect prediction of true DMCs to non-DMCs, true negatives (TN) were defined as the correct identification of non-DMCs, and false positives (FP) were defined as the incorrect prediction of true non-DMCs to DMCs. The true positive rates and false positive rates were calculated as TP/(TP + FN) and FP/(FP + TN), respectively. The results indicated that there are significant differences among methods, and these differences become large when the sequencing depth is low or when the number of replicates is small. Overall, methylSig, BSmooth, Biseq, and metilene showed lower performance than the other four, including methylKit, Fisher’s exact test, DSS, and RADMeth. Metilene could not detect DMCs, regardless of sequencing depth or the number of replicates, perhaps because metilene was initially designed for regional differential analysis. Obviously, metilene is not effective for single-CpG analysis. However, the performance of metilene was comparable to that of other methods when a regional analysis is adopted (see next section). MethylSig was the most sensitive to sequencing depth and the number of replicates, while BSmooth and Biseq were relatively stable. It is not surprising that the BSmooth and Biseq exhibited performance independent of sequencing depth and the number of replicates, since they both perform differential analysis on smoothed methylation levels. Interestingly, smoothing did not help to improve the DMC detection accuracy even for low-depth data. On the other hand, methylKit, Fisher’s exact test, DSS, and RADMeth were able to accurately identify DMCs and exhibited a similar performance when the sequencing depth was ≥15x or when the number of replicates/condition was ≥3. RADMeth performed slightly better than methylKit and DSS when the sequencing depth was low, while DSS and methylKit had a higher rate of true positives on data with a small number of replicates. Similar patterns were observed in ROC analysis on simulated datasets (Figures S1 and S2). We then evaluated each method on three bins of methylation differences (0.2–0.4, 0.4–0.6, and 0.6–0.8). The results (Tables S1 and S2) also indicated that RADMeth achieved relatively higher sensitivity, followed by methylKit. The smoothing-based approach Biseq showed the highest sensitivity on the data with small coverage, while Fisher’s exact test, DSS, and methylSig showed low sensitivity when the difference was small (0.2–04). Designing BS-seq experiments with an appropriate sequencing depth and number of replicates to maximize the benefit from the trade-off between detection power and financial cost is a common challenge. From Figure 1a,b, we can easily see that the detection power reached almost 90% on data with 5× coverage and three replicates in each condition. However, the performance could not break 60% in the absence of replicates, even with 10× coverage. This result reveals that a small number of replicates creates greater difficulty in the computational analysis of BS-seq data than does low sequencing depth, as with other sequencing applications [33,41]. Thus, including a number of biological replicates should be prioritized over obtaining more reads in BS-seq experimental design.

To avoid granting an advantage to the methods that tend to call for a large number of DMCs, we also examined the number of DMCs identified by each method (Figure 1c,d). There were also considerable differences among methods in the number of detected DMCs, which ranged from 0 to 23,011. The variations in the number of DMCs detected in data with different sequencing depths were smaller than in the data with different numbers of replicates. In most cases, the number of reported DMCs at a Benjamini–Hochberg adjusted *p*-value of 5% was less than 20,000, which is the number of gold standards, except for the number identified by Biseq in data with four replicates. The number of DMCs detected by most methods increased as the sequencing coverage or the number of replicates increased (Figure S3), except for two smoothing-based approaches, BSmooth and Biseq. The true positive rate was found to be linearly correlated with the number of detected DMCs, i.e., the higher the number of DMCs detected, the higher was the true positive rate. A noticeable exception was Biseq, which tended to select an inflated number of DMCs, resulting in a high false positive rate (Figure 1b,d).

**Figure 2:**
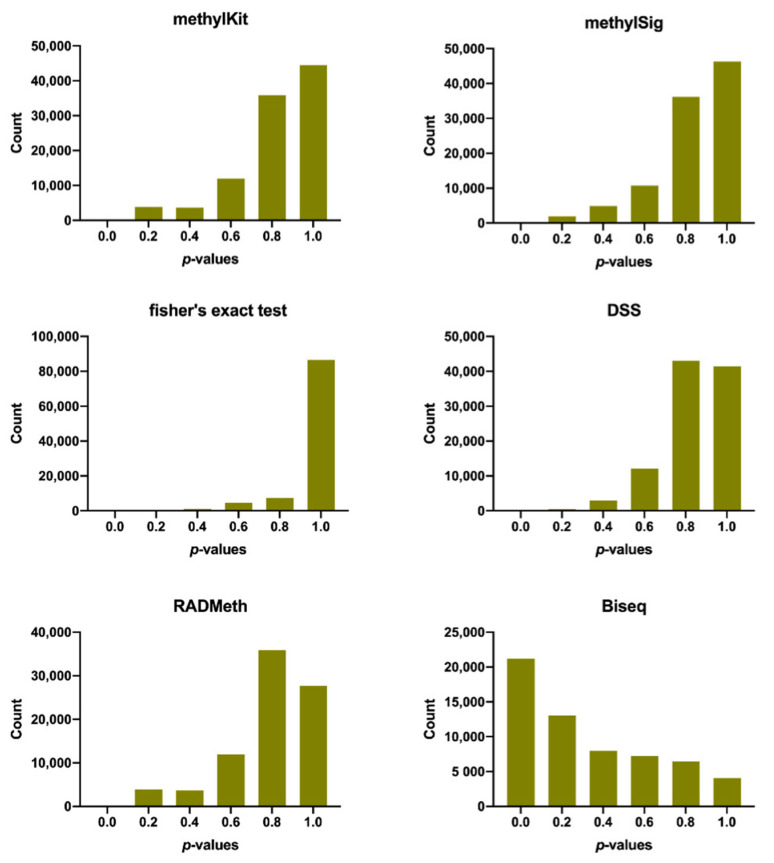
Distributions of *p*-values. Differential methylation analysis was conducted between samples from the same condition, and the *p*-values were reported. From the simulated data with 8 samples, 4 replicates belonging to same condition were extracted and randomly split into two different groups for differential analysis. Thus, no CpGs were expected to be differentially methylated in this case. BSmooth and metilene were not considered in this analysis, because their true positive rates were found to be too low in the previous analysis.

To further test the false positive rate of each method, we extracted subsamples from the simulated data to perform differential methylation analysis between samples from the same condition, and the resulting *p*-values were reported. Here, no CpGs were expected to be differentially methylated, and the *p*-values were therefore expected to be uniformly distributed. Note that BSmooth and metilene were not considered in this analysis because their true positive rates were found to be too low in the previous analysis. The number of false positives detected by each method was 37, 0, 0, 4, 36, and 15,498 for methylKit, methylSig, Fisher’s exact test, DSS, RADMeth, and Biseq, respectively. As shown in Figure 2 and Figure S4, methylSig and Fisher’s exact test correctly rejected all non-DMCs at the common significance range of <0.05, while Biseq indicated a large number of false positive predictions. Additionally, methylKit, DSS, and RADMeth showed a small number of false positives.

### 2.2. Assessment of Performance in Detecting Differentially Methylated Regions

**Figure 3:**
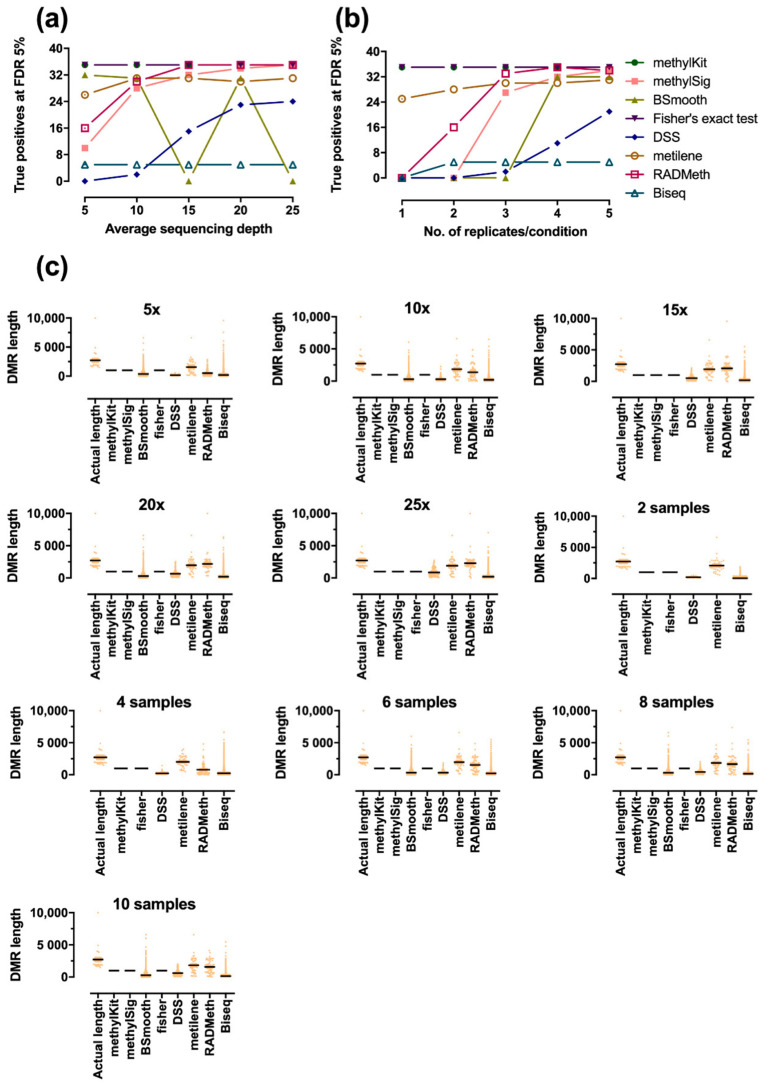
Comparison of methods for DMR detection. DMRs were identified at a 5% Benjamini–Hochberg adjusted *p*-value by each method, and the number of covered (overlapped with true positive by more than 80%) DMRs in different simulated data were reported for variations in (**a**) the average sequencing depth (3 replicates/condition in all cases) and (**b**) the number of samples (10× coverage in all cases). The distribution of DMR lengths detected by each method is summarized in (**c**). Note that we were unable to obtain the results of BSmooth with 15× and 25× coverage, since unknown execution errors occurred during the analysis.

Since the previous experiment was based on examining individual cytosines, we further examined the performances of various methods in terms of detecting DMRs on region-based simulated datasets. DMRs were extracted by each method with its own grouping strategy at a Benjamini–Hochberg adjusted *p*-value of 5%. Then, the overlapping fraction between the predicted DMRs and the true positive (Table S3) was calculated. The number of DMRs that overlapped by more than 80% was counted as covered, and the number of accurately covered DMRs of each method is shown in Figure 3a,b. The complete overlapping fractions are presented in Figures S5 and S6. From the figures, we can easily see that there were also large differences among methods in detecting DMRs. Here, methylKit and Fisher’s exact test detected all true DMRs in the different datasets. These two methods had the same results because we applied the sliding window (default window size = 1000 bp) approach implemented in methylKit to Fisher’s exact test to merge the CpGs into regions, since Fisher’s exact test itself does not include a regional analysis function. RADmeth and methylSig had comparable performance when the sequencing depth was ≥15× or when the number of replicates/condition was ≥3. However, the performance decreased dramatically when the coverage or the number of replicates was small. In addition, metilene achieved markedly better performance in regional analysis compared to that in DMC analysis. Metilene was found to be the approach that most accurately identified exact DMR boundaries, as shown in Figure 3c, which summarizes the length distributions of the detected DMRs. RADMeth could also accurately identify DMR boundaries when the sequencing depth and the number of replicates is high. On the other hand, the DMRs found in methylKit were on average shorter than the gold standard, indicating that methylKit could correctly cover true DMRs, but that metilene could more accurately detect DMR boundaries. MethylKit segments the genome using sliding windows, while metilene merges CpGs based on their actual genomic position. Interestingly, the detection power of BSmooth was also substantially better in DMR analysis, except in specific cases, such as in 15× and 25× coverage, where an unknown execution error occurred during analysis. DSS and Biseq were clearly more suitable for DMC analysis.

### 2.3. Differential Analysis of Mouse Methylome

**Figure 4:**
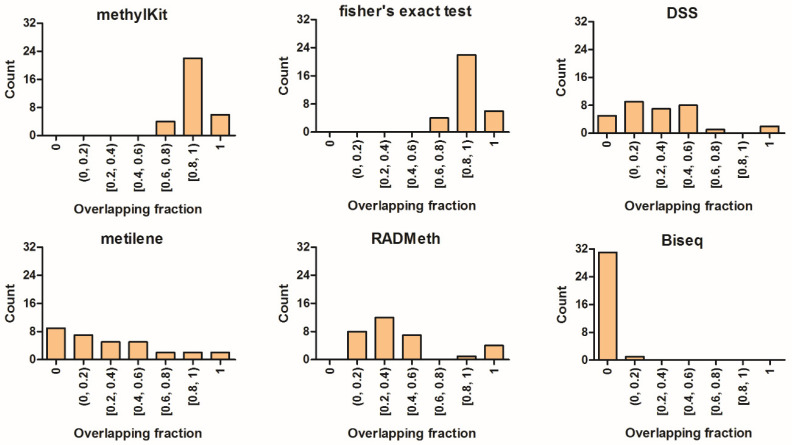
Histogram of the overlapping fraction for mouse methylome data. DMRs were identified at a 5% Benjamini–Hochberg adjusted *p*-value by each method, and the overlapping fraction (length of overlapping region/length of gold standard) between the gold standard and detected DMRs was calculated using BEDtools [45]. Thus, a value of 1 indicates perfect overlap, while a value of 0 indicates no common regions. Note that the results of methylSig and BSmooth are absent because the data contained no replicates.

To test the methods on real biological data, we used Xie’s [43] whole-genome bisulfite sequencing data from the mouse methylome to perform differential analysis. Their study reported 55 parent-of-origin-dependent DMRs (32 known imprinted DMRs +23 novel DMRs). We used the 32 biologically verified DMRs (Table S4) as the gold standard for method evaluation, as in [44,45]. The overlapping fraction between the gold standard and DMRs detected by each method was reported (Figure 4). Similar results were obtained on the mouse data and the simulated data. MethylKit and Fisher’s exact test had identical results and covered all 32 gold standard DMRs with greater than 60% overlap (six with complete overlap and 22 with greater than 80% overlap). RADMeth also covered all the true positives, but with a relatively lower overlapping fraction than methylKit and Fisher’s exact test. DSS and metilene exhibited an overall similar performance, in which metilene had more overlaps (>60%) than DSS, while DSS covered more DMRs than metilene. As in the regional simulation experiment, Biseq failed to detect most DMRs.

### 2.4. Differential Analysis of the Human Methylome

**Figure 5:**
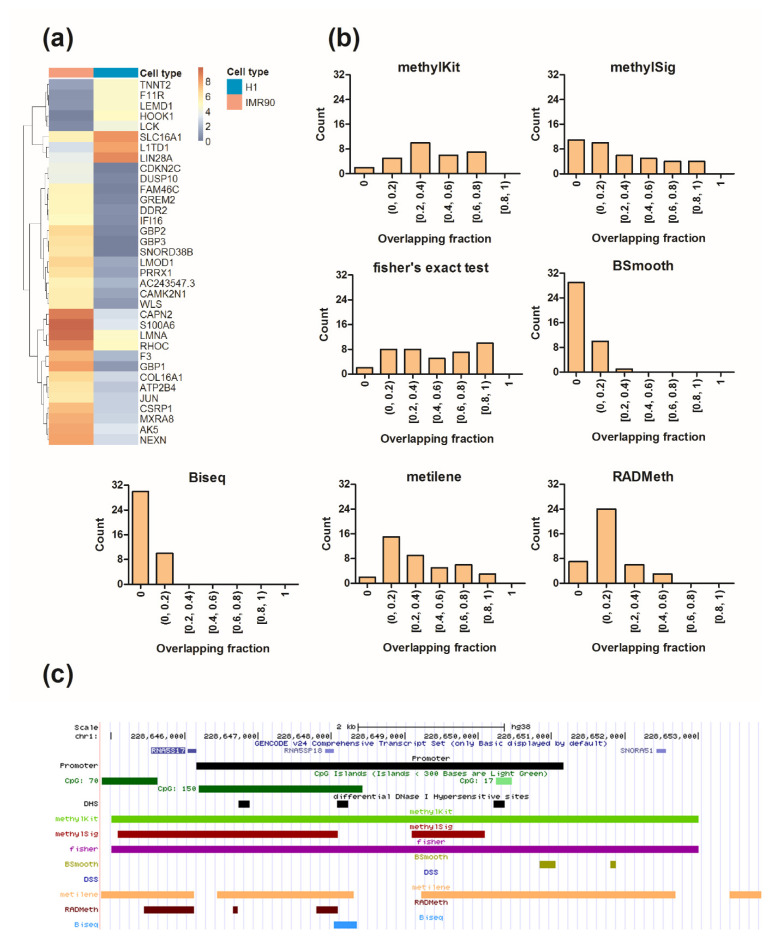
DMR analysis results for human methylome data. DEGs between IMR90 and H1-hESC cells were called at an adjusted *p*-value < 0.05 and >2-fold change, and the heatmap is shown in (**a**). DMRs were identified at a 5% Benjamini–Hochberg adjusted *p*-value by each method, and the overlapping fraction (**b**) (length of overlapping region/length of gold standard) between the gold standard and the detected DMRs was calculated using BEDtools [45]. The genomic view of the RNA5S17 gene, upstream of TSS, CpG islands, differential DHSs, and DMRs reported by each method are shown in (**c**).

To evaluate the performance of each method on human BS-seq data, we conducted a differential methylation analysis between IMR90 human lung fibroblasts (IMR90) and H1 human embryonic stem cells (H1-hESCs). DMRs were again extracted by each method with its own grouping method at a Benjamini–Hochberg adjusted *p*-value of 5%. As the true DMRs were unknown, we employed RNA-seq and DNase-seq data on the IMR90 and H1-hESC cell lines to infer the gold standard. Gene expression has been found to be regulated by DNA methylation [46,47], and the promoter regions of different cell types have shown considerably distinct methylation patterns [48,49]. Thus, the promoter regions of differentially expressed genes (DEGs) tend to be differentially methylated, which can serve as the gold standard for benchmarking. We performed differential expression analysis between IMR90 and H1-hESC cells using ENCODE [50] RNA-seq data (see Materials and Methods). The results identified 505 significant DEGs (Table S5), and GO analysis revealed significant enrichment in genes involved in extracellular matrix organization, cell adhesion, response to drugs, and collagen catabolic process (*p* = 1  ×  10^−9^, Table S6). We note that the promoter regions of 61 DEGs in chromosome 1 were chosen for the downstream gold standard construction for ease of illustration, and the heatmap of these genes is shown in Figure 5a. DNA methylation and chromatin accessibility are well known to be highly correlated with each other, rather than being independent [51,52]. In general, a gene is expressed if its promoter region remains in an open chromatin and unmethylated state, while a gene is silenced if it has a closed and methylated promoter [7,53]. Thus, promoters with differential chromatin configurations are more likely to be differentially methylated. Accordingly, we first extracted the DNase I hypersensitive sites (DHSs) of IMR90 and H1-hESC cells identified in ENCODE and carried out a differential analysis between those peaks. Finally, we selected the promoters of DEGs that contained differential DHSs as the gold standard. The overlapping fraction between the gold standard and the DMRs detected by each method on chromosome 1 was reported in Figure 5b using BEDTools [45]. The results on other chromosomes were reported in Figure S7. The UCSC genome browser [54] displays of the detected DMRs and various genomic regulatory regions, including 5 kb upstream of the transcription start site of the example gene RNA5S17, CpG islands, and differential DHSs, are shown in Figure 5c. The DMRs detected by methylKit, methylSig, Fisher’s exact test, and metilene were located across various genomic regions, including promoter, gene body, UTRs, and intergenic regions, while the DMRs detected by Biseq and BSmooth were located in promoter and intergenic regions. Consistent with previous results, methylKit, Fisher’s exact test, and metilene covered most DMRs, except two that were CpG-sparse regions. RADMeth also identified a reasonable number of DMRs, but the overall overlapping fractions were slightly lower than those of the above three methods. Unfortunately, two smoothing-based approaches, BSmooth and Biseq, detected only a small number of DMRs, and DSS surprisingly failed to detect DMRs.

## 3. Conclusions

We have presented a detailed comparative analysis of a number of computational approaches for identifying differentially methylated cytosines/regions from bisulfite sequencing data. Our analysis focused on the performance of each method in terms of the true positive rate, effect of sequencing depth and the number of replicates on differential analysis, false positives on a null model, DMR boundaries, and performances in omic analysis. Overall, there were notable variations among methods, and no single method consistently performed best in all benchmarking. For DMC analysis, RADMeth, methylKit, DSS, and Fisher’s exact test had comparable performance when the coverage or the number of replicates was high enough. However, RADMeth and methylKit clearly had better sensitivity on data with a low sequencing depth, while DSS and methylKit had better sensitivity on data without replicates. For DMR analysis, methylKit and Fisher’s exact test covered more DMRs than other methods, and metilene performed especially well in identifying correct DMR boundaries. Interestingly, smoothing-based approaches did not greatly improve the performance of differential analysis. Additionally, a small number of replicates presented more difficulties in computational analysis of BS-seq data than low sequencing depth did. This finding suggests that including a number of biological replicates should be prioritized over obtaining more reads in BS-seq experimental design. Data analysis and interpretation should be performed with great care, especially when the number of replicates or sequencing depth is limited. Moreover, it is difficult to cross-use methods in different tools, because they have large differences in data structures and programming languages. More efforts are needed for developers to simplify the data analysis procedure and enable integrated analysis of methods in different tools or software packages. 

In summary, as sequencing technology advances, BS-seq data analysis will continue to be a major issue for computer scientists and biologists. To the best of our knowledge, this study is the first comprehensive comparison of commonly used differential methylation analysis methods on both synthetic and real data. There are also some open questions we did not consider in this research, such as performance on 5 hmC and single cell BS-seq experiments and the effect of different parameters. We expect our study to be a valuable resource for choosing an appropriate BS-seq data analysis method and a helpful direction for future tool development.

## 4. Materials and Methods

### 4.1. Simulation

To assess the ability of the various methods to detect DMCs, we randomly extracted 1 million CpG sites from the Lister’s IMR90 bisulfite sequencing data [22] and simulated 20% of them as true positives and all others as true negatives. The methylation difference of each true positive was randomly assigned within the range of 0.2 to 1, corresponding to weak and strong differences, respectively. Using above strategies, we generated five datasets with different average sequencing depths (5×, 10×, 15×, 20×, and 25×) and five datasets with a different number of biological replicates per condition (1, 2, 3, 4, and 5). To assess the performance of the methods in region-based DMR detection, we further simulated five datasets with different sequencing depths (5×, 10×, 15×, 20×, and 25×) and five datasets with a different number of replicates (1, 2, 3, 4, and 5). Taking 35 DMRs found in Lister’s study [22] as the gold standard true positives, we randomly assigned the methylation differences of the CpGs within the true positive regions from 0.2 to 1, while other CpGs within the remaining genome were simulated with no difference between groups.

### 4.2. RNA-Seq

The TPM (transcripts per million) normalized count of RNA-seq data for IMR90 (accession: ENCFF833OTW) and H1-hESCs (accession: ENCFF093NEQ) were obtained from ENCODE [50]. An Illumina Genome Analyzer II was used to sequence mRNAs isolated from IMR90 and H1-hESCs, and the produced reads were mapped to the human reference genome using Pash. Differential analysis of RNA-seq data was performed using the edgeR [55] software package, and DEGs were detected with an adjusted *p*-value < 0.05 and >2-fold change. The genomic coordinates of the DEGs were extracted based on human genome assembly (GRCh38) using Ensembl Biomart [56], and the promoter regions were defined as the 5 kbp up/downstream of transcription start sites. 

### 4.3. DNase-Seq 

The DNase-seq data of IMR90 (accession: ENCFF136QTV) and H1-hESCs (accession: ENCFF184VRJ) were also downloaded from ENCODE, and the reads were also sequenced on an Illumina Genome Analyzer II. Downstream data analysis followed the ENCODE guidelines, and the narrow peaks called from the ENCODE were directly used for the differential analysis. The differential DHSs between IMR90 and H1 were identified using BEDTools [45].

### 4.4. BS-Seq

The BS-seq data of IMR90 and H1 were obtained from the human DNA methylome database of Salk Institute [57]. Four samples (two replicates each) were sequenced on an Illumina Genome Analyzer II with an average depth of 14.5× per strand, yielding 1.16 and 1.18 billion reads for IMR90 and H1, respectively. The reads were originally mapped to the hg18 reference genome, covering 2464,851 CpGs in chromosome 1. The genome coordinates were then converted from hg18 to hg38 using the UCSC liftOver command line version. The mouse BS-seq data [43] were downloaded from the Gene Expression Omnibus (accession: GSE33722). Two reciprocal crosses were sequenced on an Illumina Genome Analyzer II with an average depth of 23.75×, generating 1.54 billion and 1.33 billion reads for F1i and F1r, respectively. The reads were mapped to the mm9 reference genome, covering 11,345,372 CpG dinucleotides.

## Figures and Tables

**Table 1:** A brief summary of tools for benchmarking.

Tool	Version	Model Assumption	Differential Methylation Test	Segmentation	Language	Smoothing
Fisher’s	1.8.2	-	Fisher’s exact test	tilling window	R	No
BSmooth	1.8.2	binomial distribution	modified t-test	merging consecutive CpGs	R	Yes
methylKit	0.99.2	logistic regression	logistic regression test	tilling window or predefined regions	R	No
methylSig	0.4.4	beta-binomial model	likelihood ratio test	tilling window	R	No
DSS	2.12.0	Bayesian hierarchical model	Wald test	merging CpGs based on *p*-value	R	No
metilene	0.2–6	Nonparametric method	2D Kolmogorov–Smirnov	circular binary segmentation	C	No
RADMeth	-	beta-binomial regression	log-likelihood ratio test	correlation between *p*-value pairs within a bin	C++	No
Biseq	1.12.0	Beta regression model	Wald test	merging consecutive CpGs	R	Yes

## Data Availability

Publicly available datasets were analyzed in this study. This data and execution codes can be found here [58].

## Supplementary Materials

The following are available online at https://www.mdpi.com/article/10.3390/ijerph18157975/s1, Figure S1: ROC analysis by changing adjusted p-value cutoffs (0.001, 0.005, 0.01, 0.05) on simulated datasets with different sequencing depth, Figure S2: ROC analysis by changing adjusted p-value cutoffs (0.001, 0.005, 0.01, 0.05) on simulated datasets with different number of replicates, Figure S3: The number of detected differentially methylated cytosines by each method (a) varying the sequencing depth and (b) varying the number of replicates, Figure S4: Evaluation of false positives under null model, Figure S5: Overlapping fraction on simulated datasets with different sequencing depth, Figure S6: Overlapping fraction on simulated datasets with different number of replicates, Figure S7: Overlapping fraction on IMR data between gold standard and DMRs found by each method, Table S1: Performances for three bins of methylation difference on simulated datasets with different coverages, Table S2: Performances for three bins of methylation difference on simulated datasets with different number of samples, Table S3: 35 gold standard DMRs for region-based simulation, Table S4: 32 biologically verified DMRs from Xie’s data, Table S5: DEGs bewteen IMR90 and H1-hESC cell.
