# Supplementary material for: Comprehensive Evaluation of Differential Methylation Analysis Methods for Bisulfite Sequencing Data
Source: Int J Environ Res Public Health. 2021 Jul 28;18(15):7975. doi: 10.3390/ijerph18157975 (PMC8345583; doi:10.3390/ijerph18157975)
Supplement: Supplementary file 1 [file ijerph-18-07975-s001.zip › ijerph-1235340-supplementary.pdf]

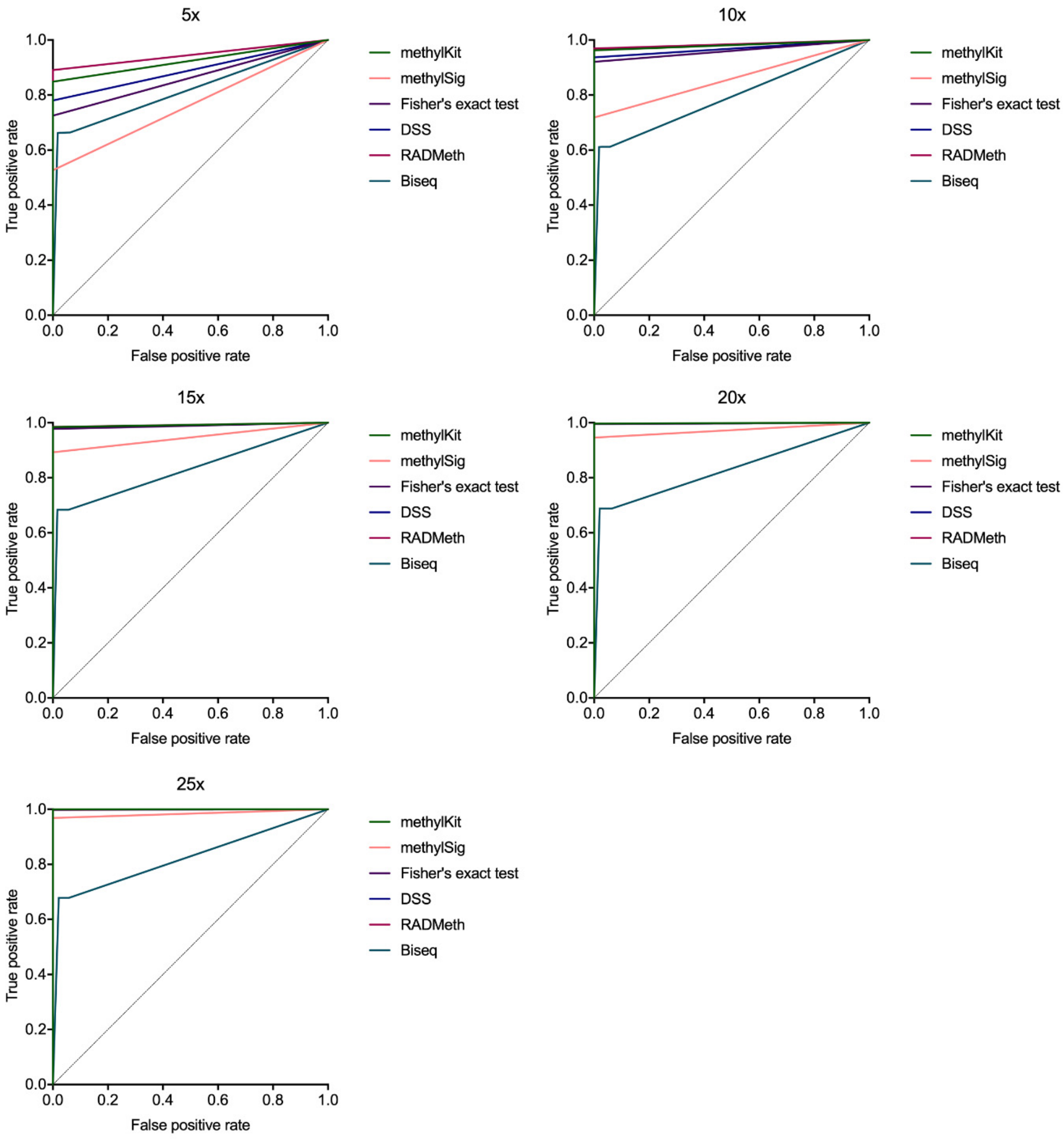

Figure S1. ROC analysis by changing adjusted *p*-value cutoffs (0.001, 0.005, 0.01, 0.05) on simulated datasets with different sequencing depth. Most methods revealed best performances on 0.05 cutoffs while Biseq showed better performance on smaller cutoff.

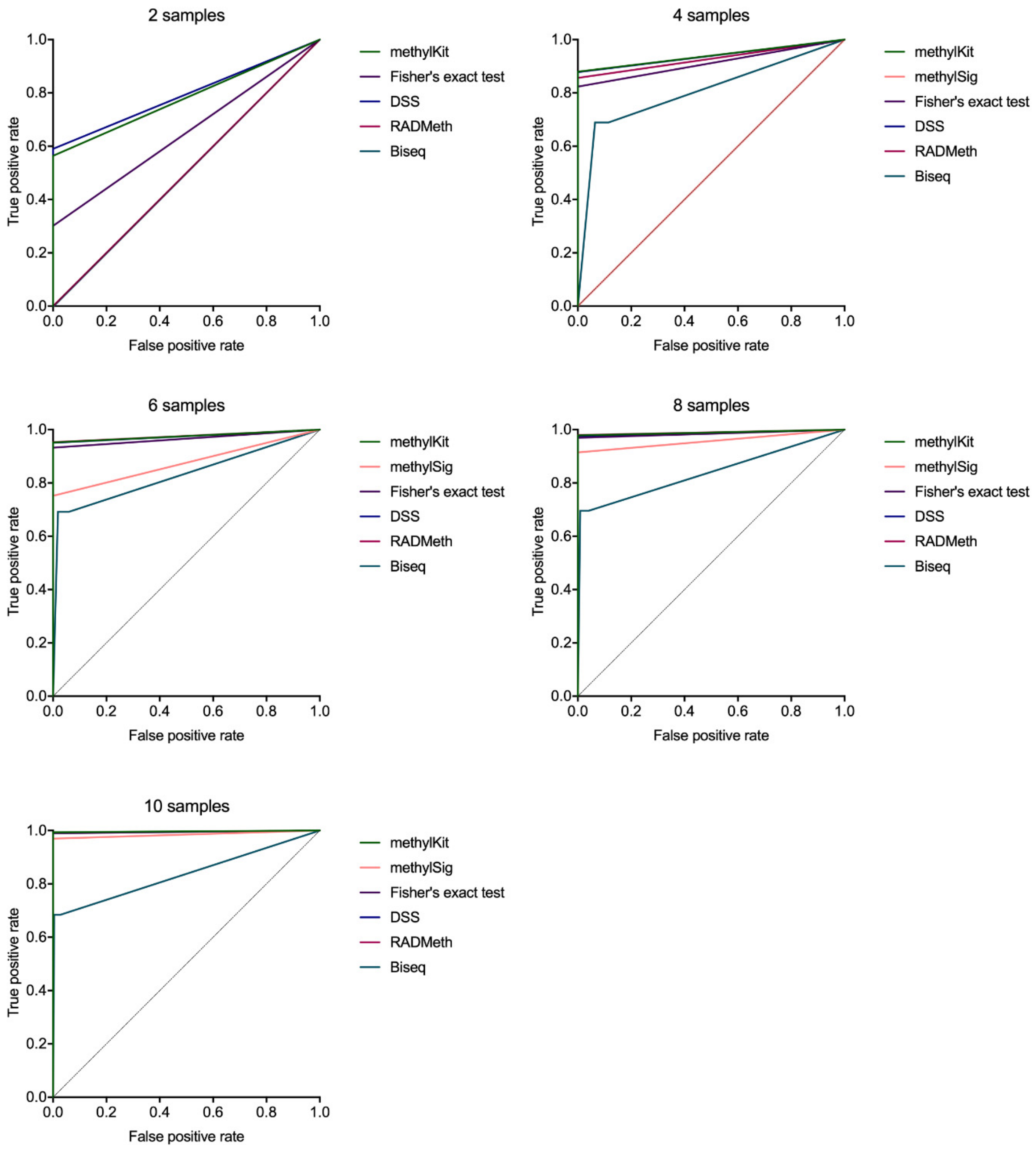

Figure S2. ROC analysis by changing adjusted *p*-value cutoffs (0.001, 0.005, 0.01, 0.05) on simulated datasets with different number of replicates. Most methods revealed best performances on 0.05 cutoffs while Biseq showed better performance on smaller cutoff.

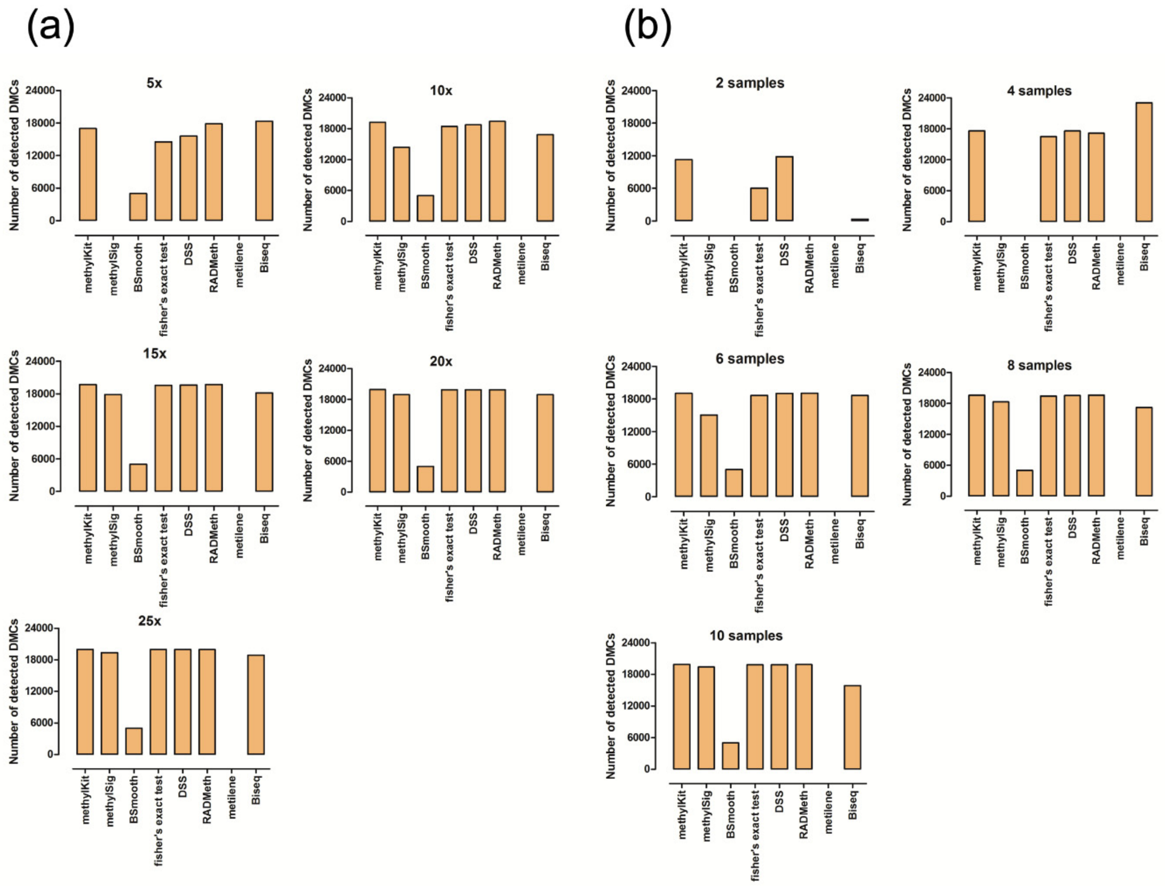

Figure S3. The number of detected differentially methylated cytosines by each method (a) varying the sequencing depth and (b) varying the number of replicates.

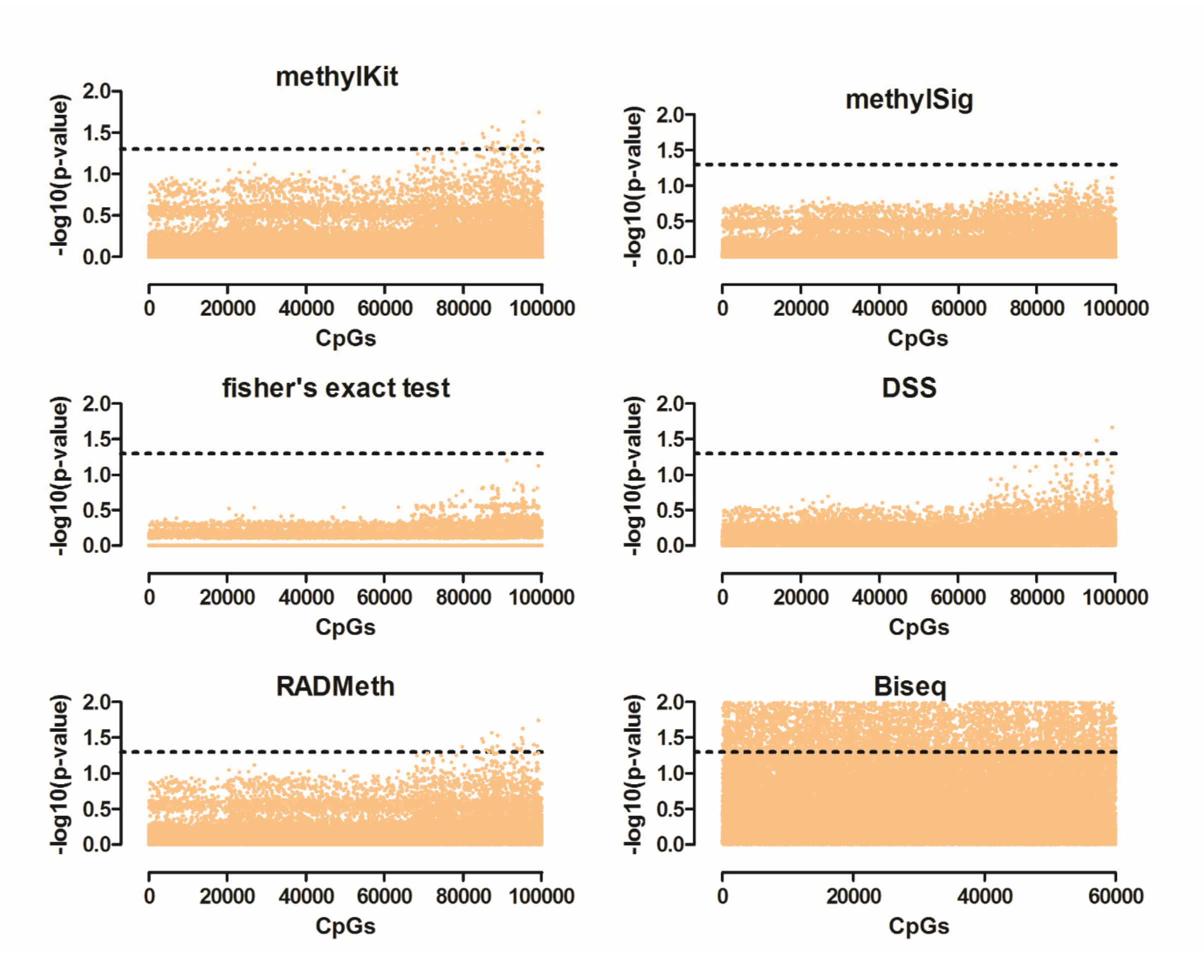

Figure S4. Evaluation of false positives under null model. Differential methylation analysis was conducted between samples from the same condition, and log-transformed p-values were reported. From the simulated data with 8 samples, 4 replicates belong to same condition were extracted and randomly split into two different groups for differential analysis. Thus, no CpGs are expected to be differentially methylated in this case. The dashed line indicates the common significance range of < 0.05. BSmooth and metilene were not considered in this analysis because their true positive rates were found to be too low in the previous analysis.

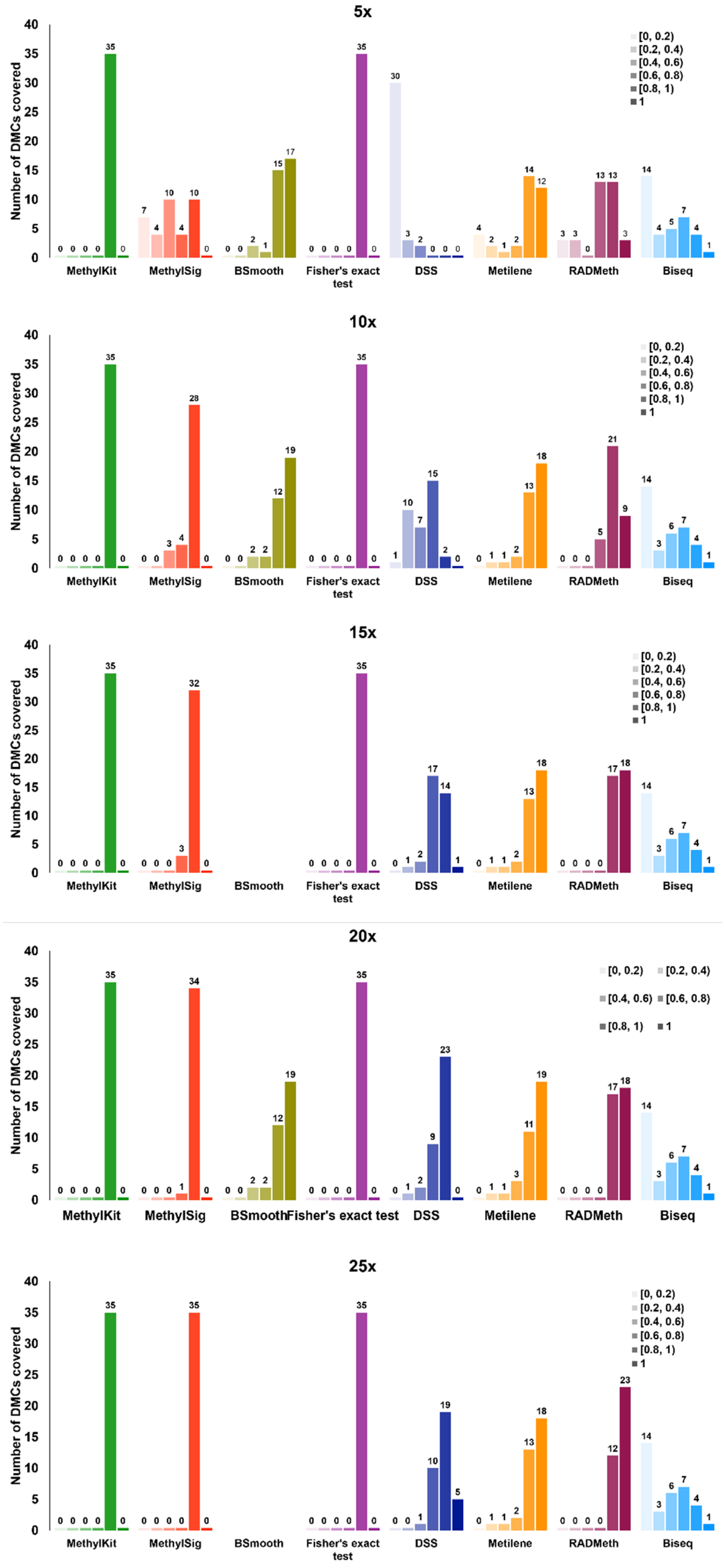
5x
10x
15x
20x
25x
Number of DMCs covered
[0, 0.2)
[0.2, 0.4)
[0.4, 0.6)
[0.6, 0.8)
[0.8, 1)
1
MethylKit
MethylSig
BSmooth
Fisher's exact test
DSS
Metilene
RADMeth
Biseq

Figure S5. Overlapping fraction on simulated datasets with different sequencing depth. The overlapping fraction was calculated as the length of overlapping region between gold standard and detected DMRs divided by the length of gold standard. Thus, a value of 1 indicates perfect overlap while a value of 0 means no common regions. For each method, the number of DMRs are summarized at 6 overlap bins 0 - 20%, 20 - 40%, 40% - 60%, 60% - 80%, 80% - 100%, and 100%.

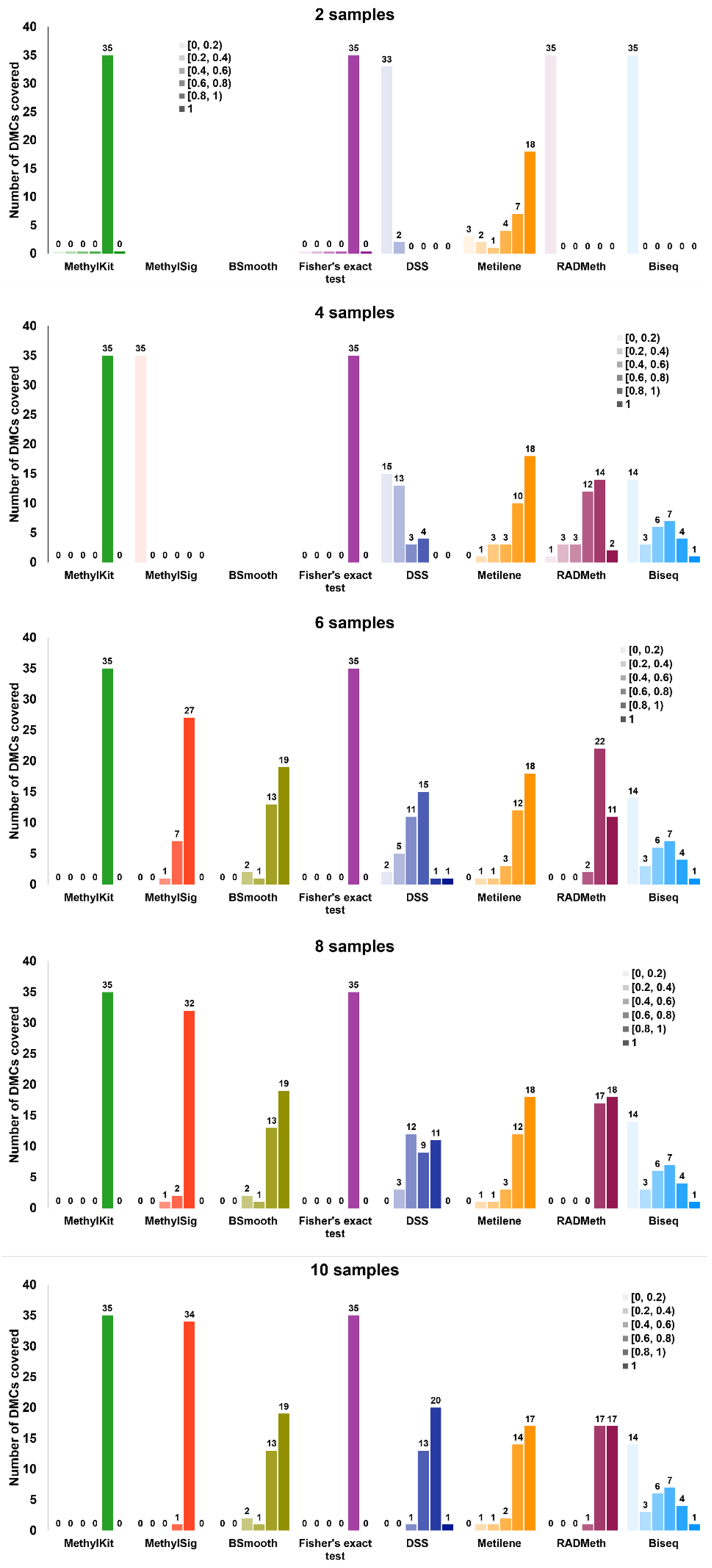
2 samples
4 samples
6 samples
8 samples
10 samples
Number of DMCs covered
[0, 0.2)
[0.2, 0.4)
[0.4, 0.6)
[0.6, 0.8)
[0.8, 1)
1
MethylKit
MethylSig
BSmooth
Fisher's exact test
DSS
Metilene
RADMeth
Biseq

Figure S6. Overlapping fraction on simulated datasets with different number of replicates. The overlapping fraction was calculated as the length of overlapping region between gold standard and detected DMRs divided by the length of gold standard. Thus, a value of 1 indicates perfect overlap while a value of 0 means no common regions. For each method, the number of DMRs are summarized at 6 overlap bins 0 - 20%, 20 - 40%, 40% - 60%, 60% - 80%, 80% - 100%, and 100%.

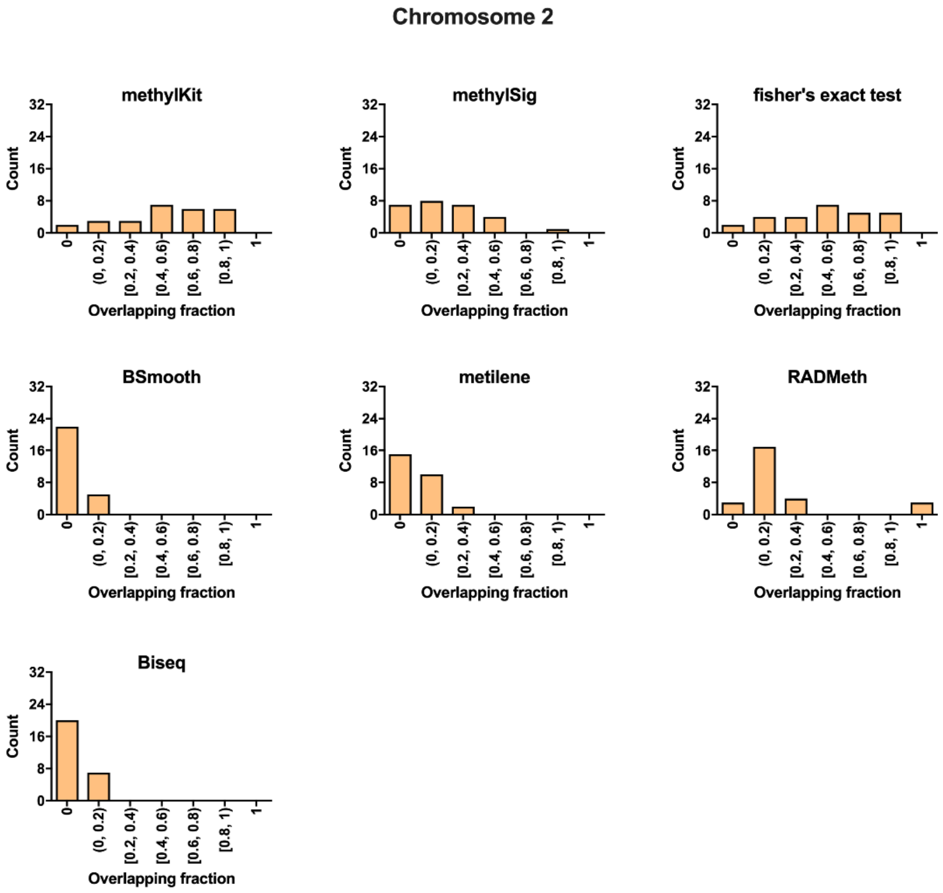

Chromosome 3

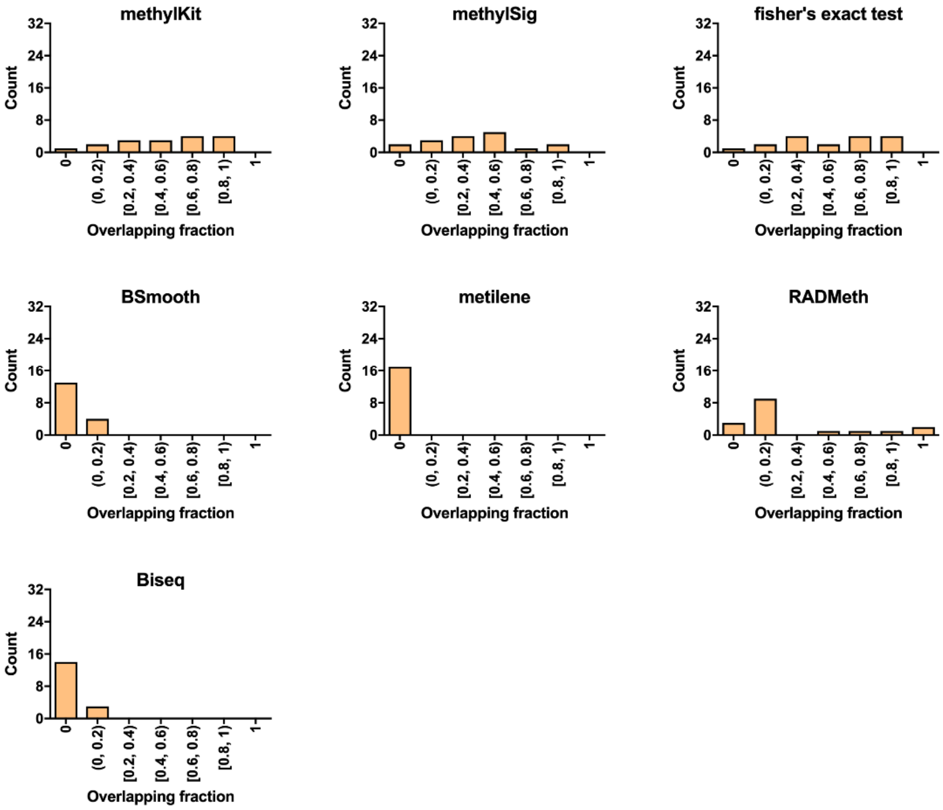

Chromosome 4

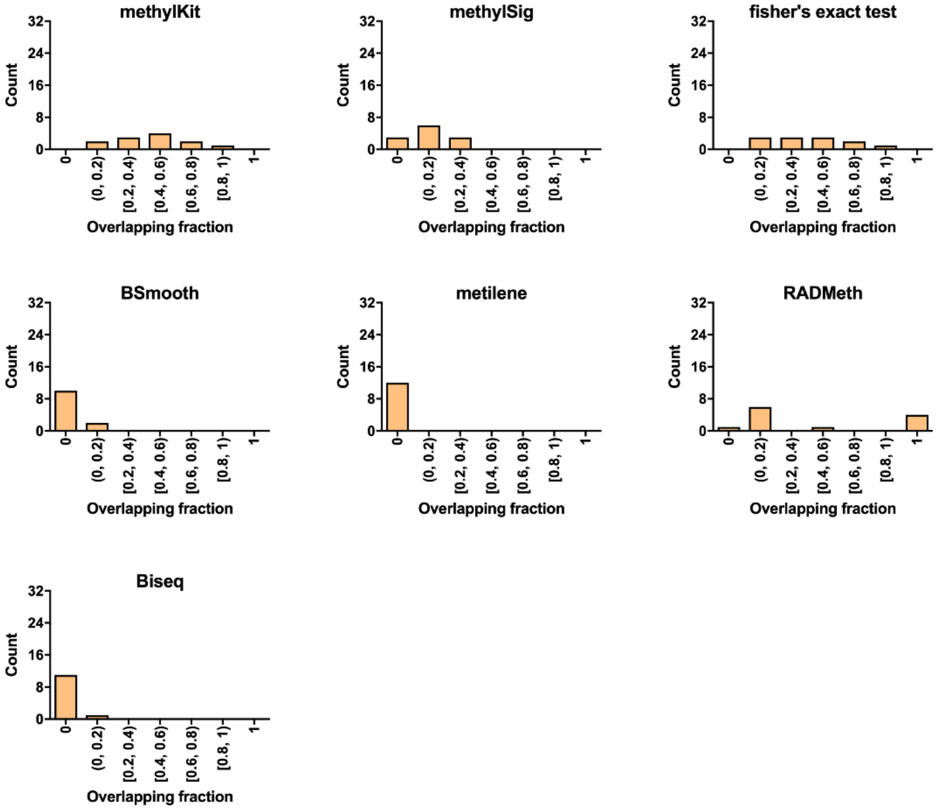

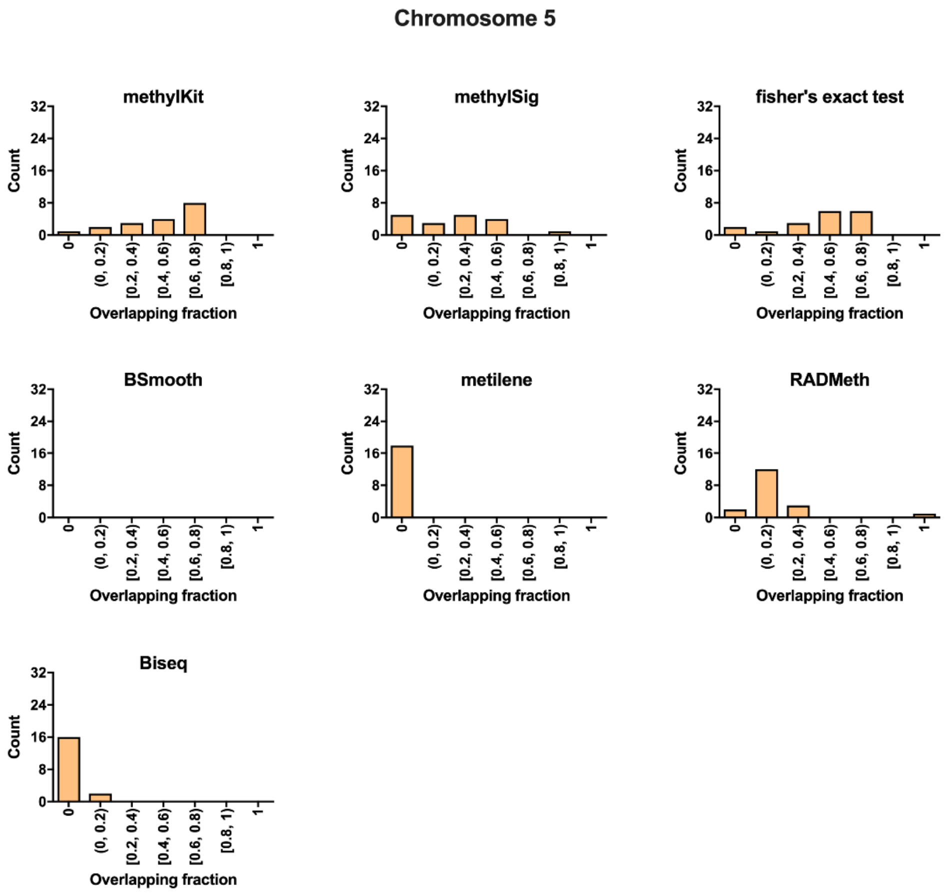
Chromosome 5
methylKit
methylSig
fisher's exact test
BSmooth
metilene
RADMeth
Biseq
Count
32
24
16
8
0
0
(0, 0.2)
[0.2, 0.4)
[0.4, 0.6)
[0.6, 0.8)
[0.8, 1)
1
Overlapping fraction

Chromosome 6

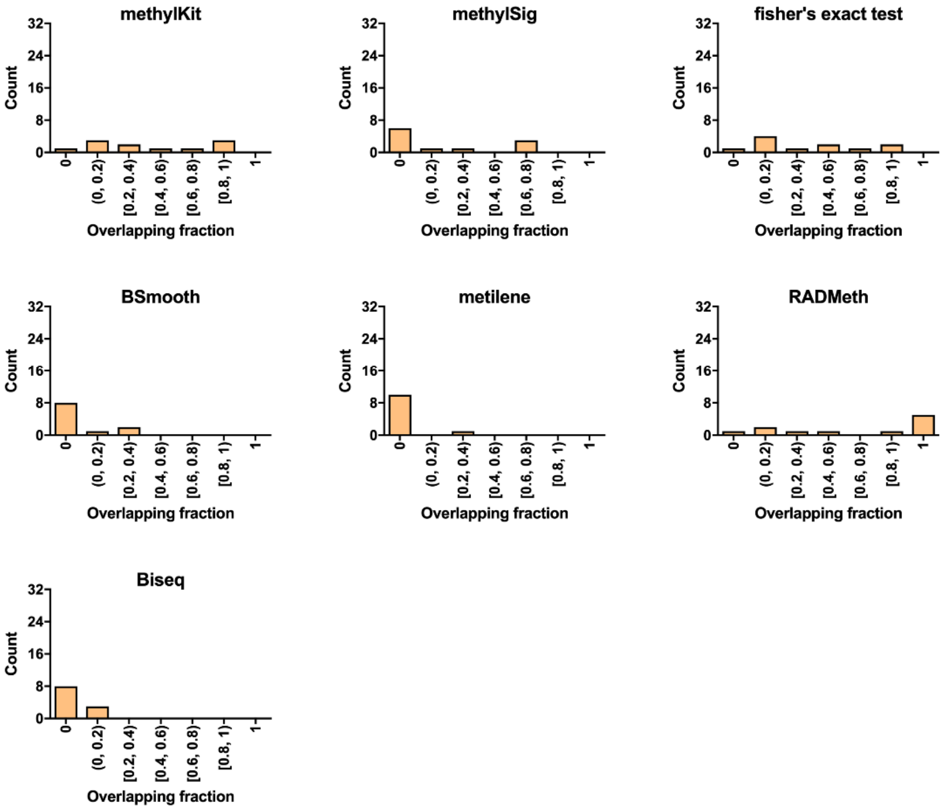

Chromosome 7

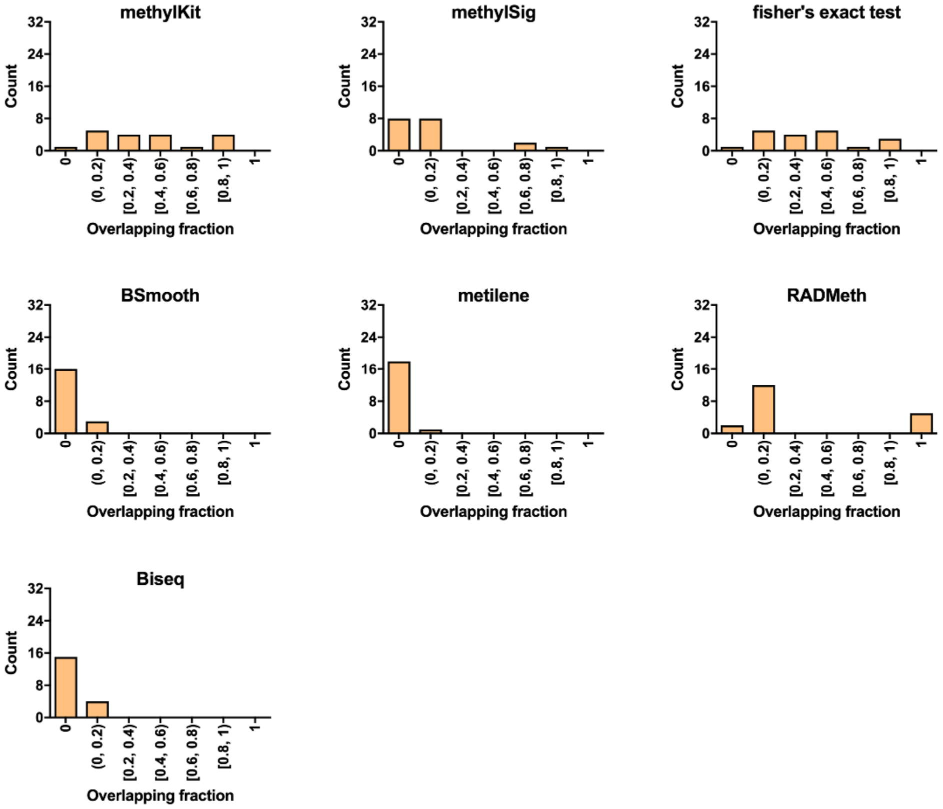

Chromosome 8

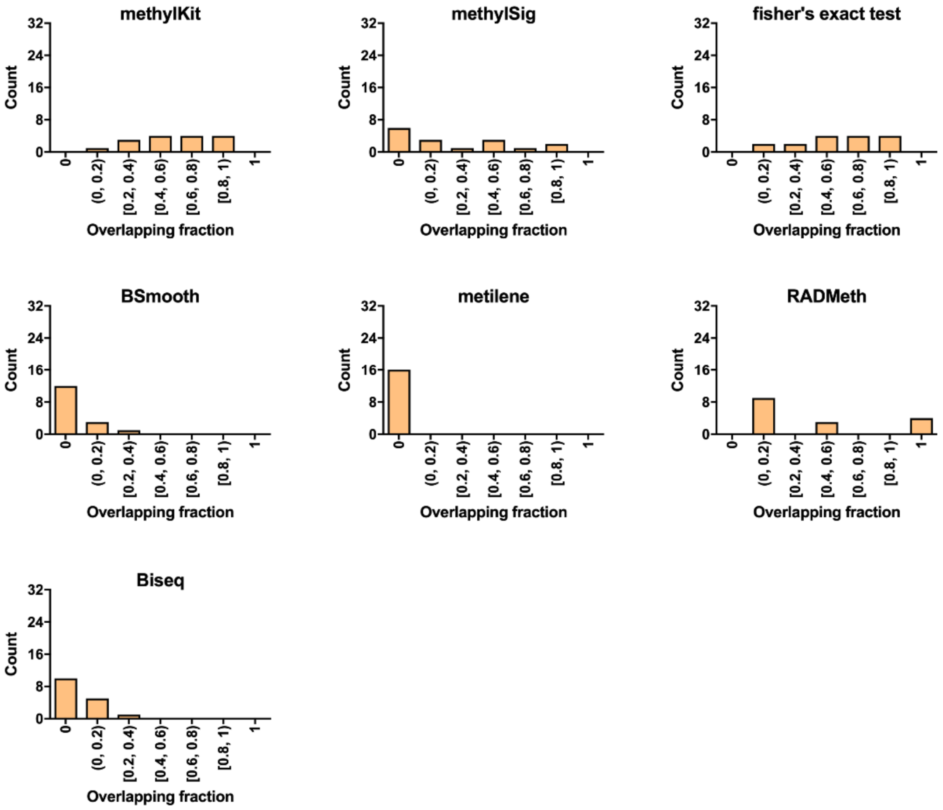

Chromosome 9

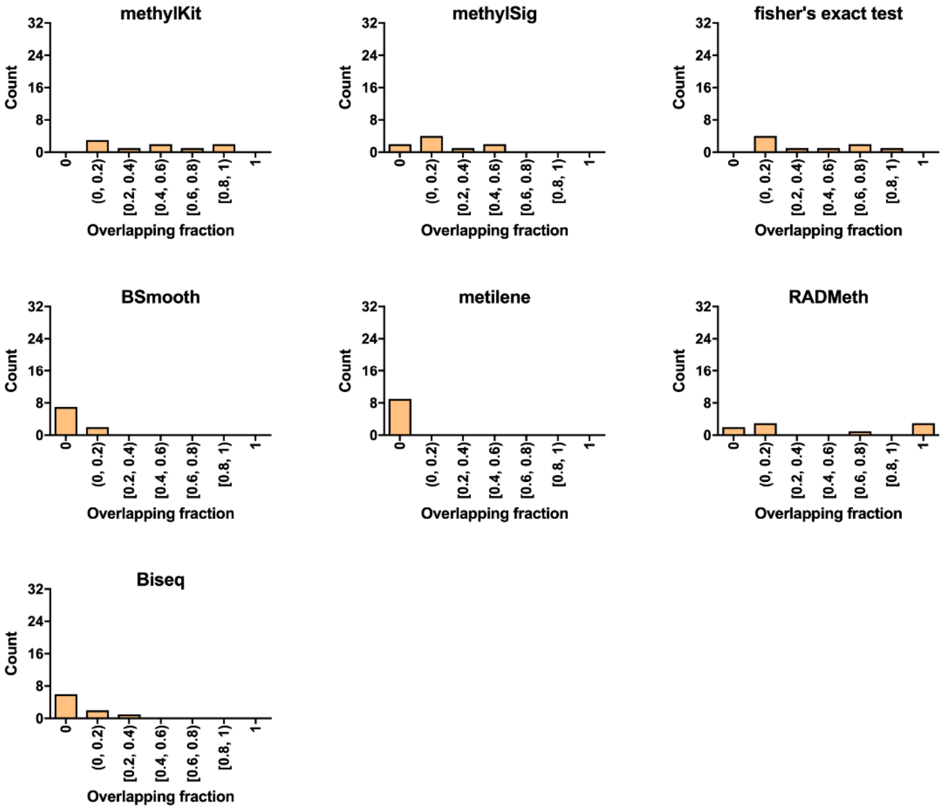

Chromosome 10

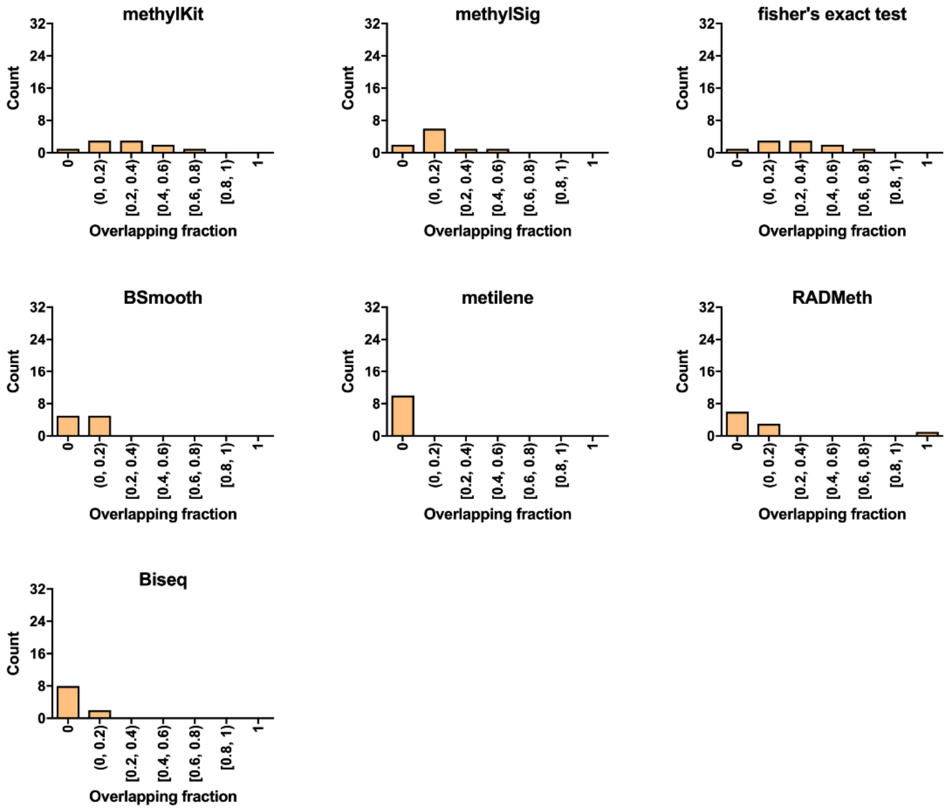

Chromosome 11

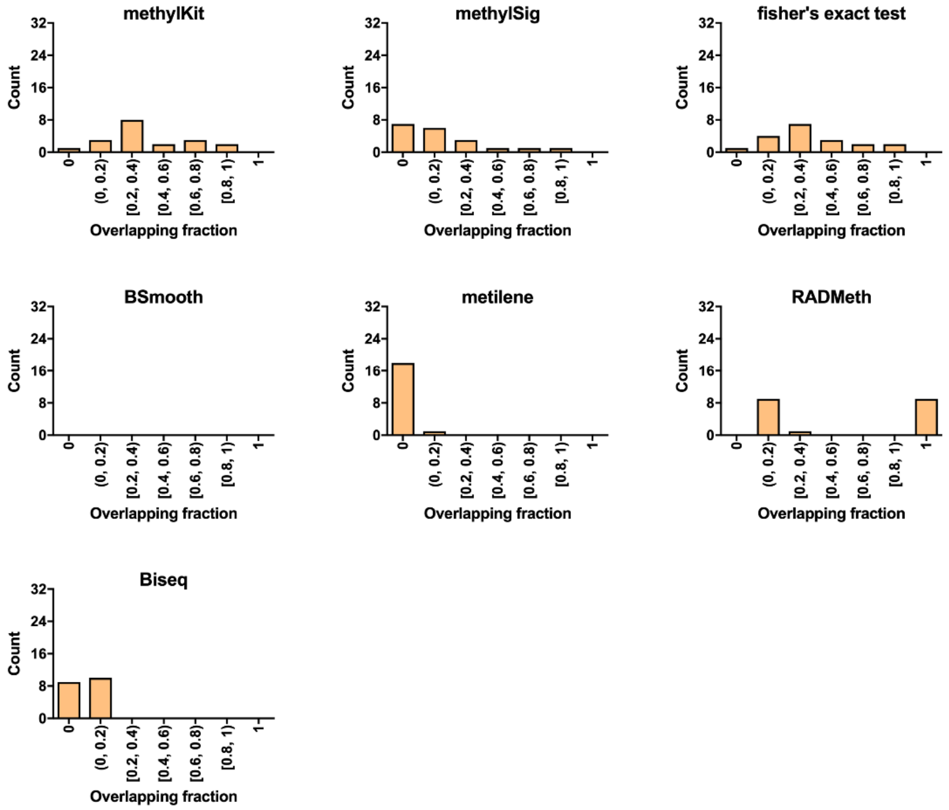

Chromosome 12

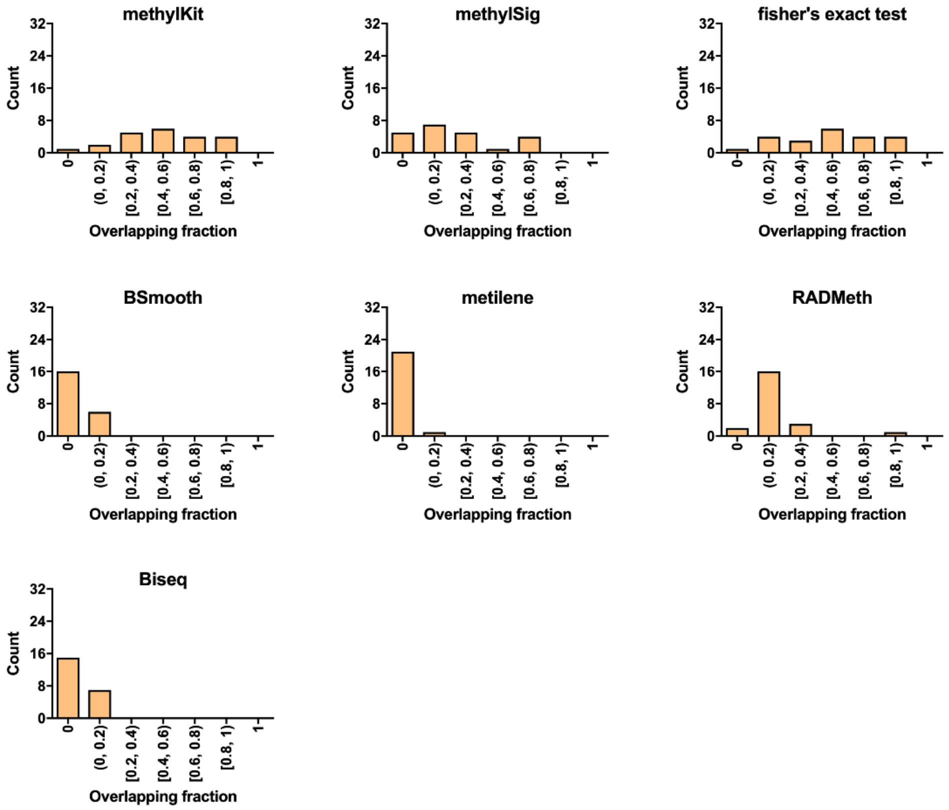

Chromosome 13

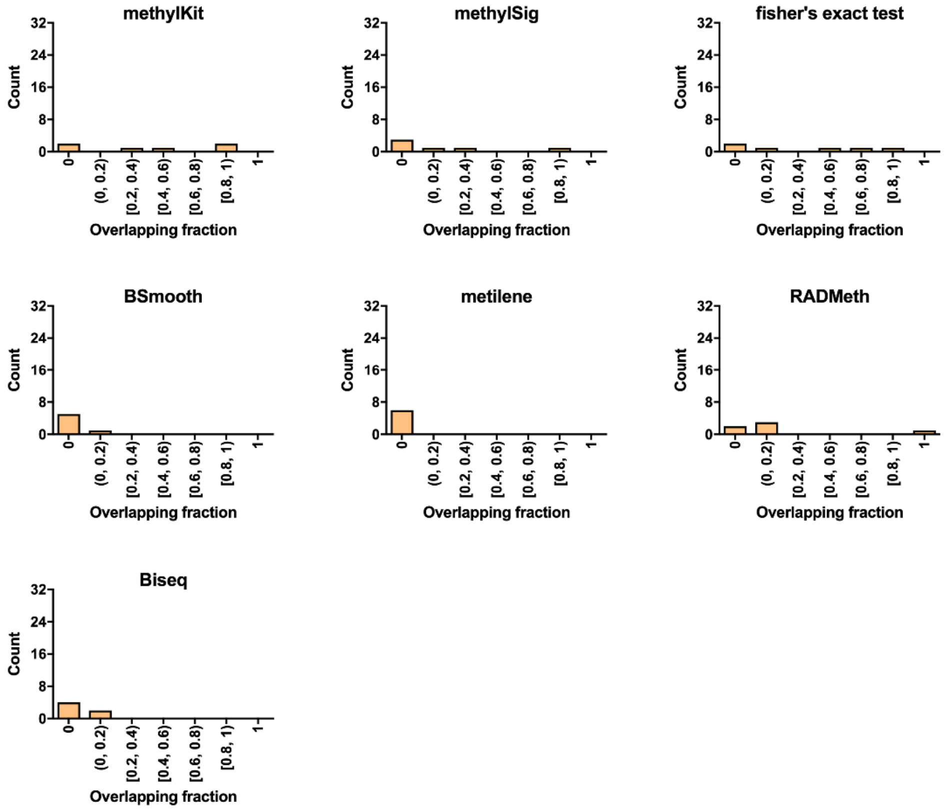

Chromosome 14

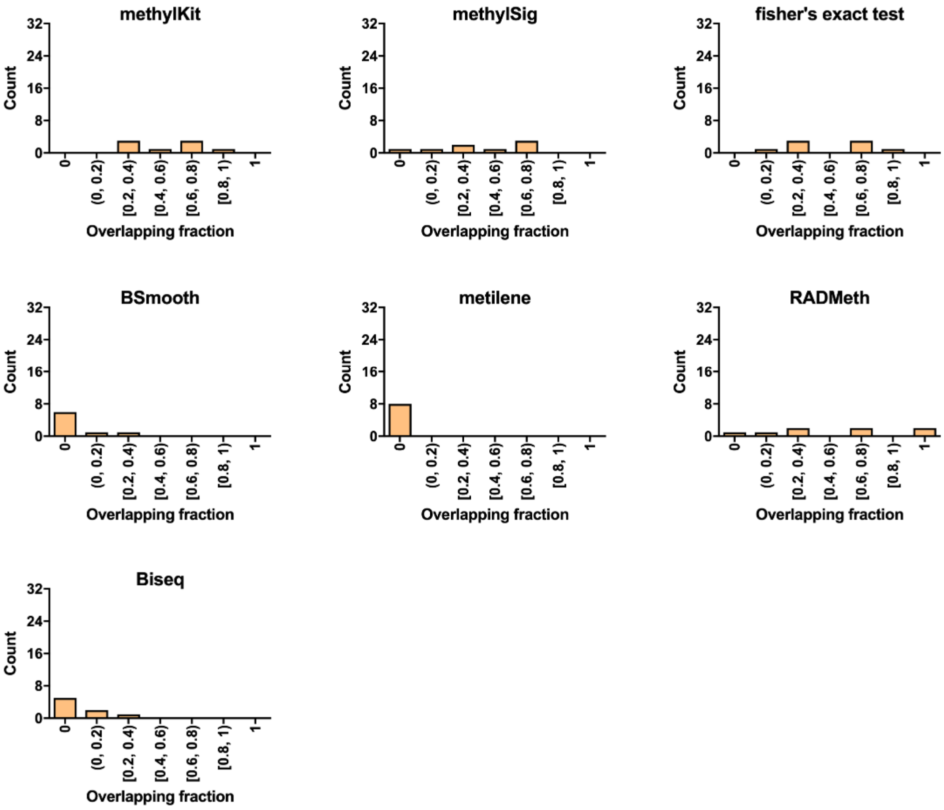

Chromosome 15

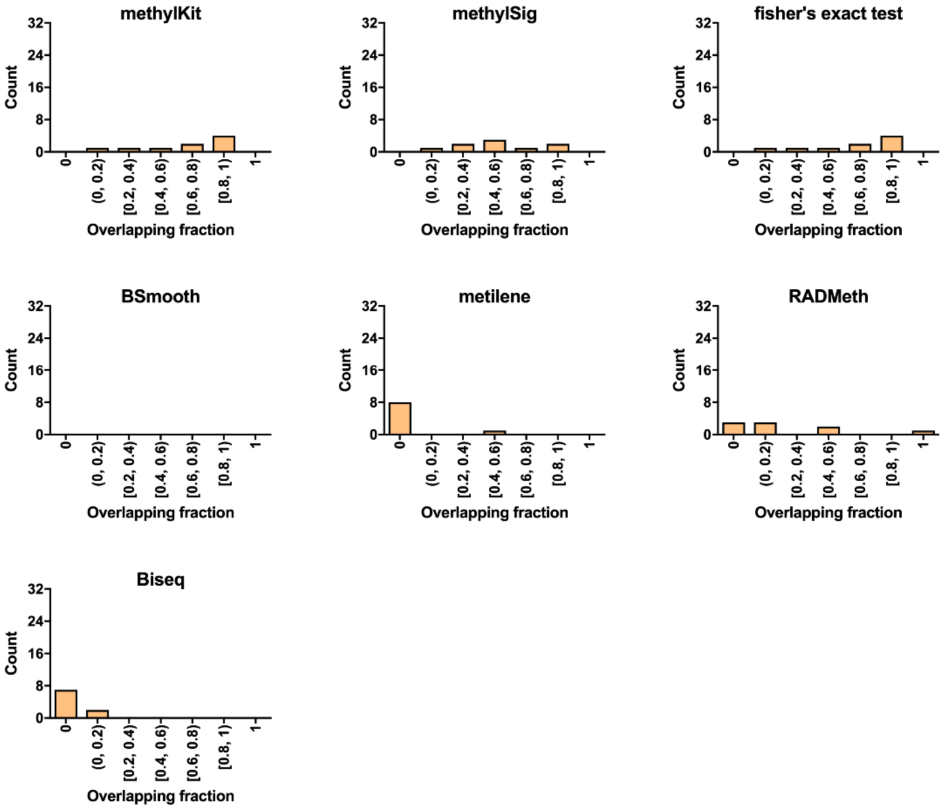

Chromosome 16

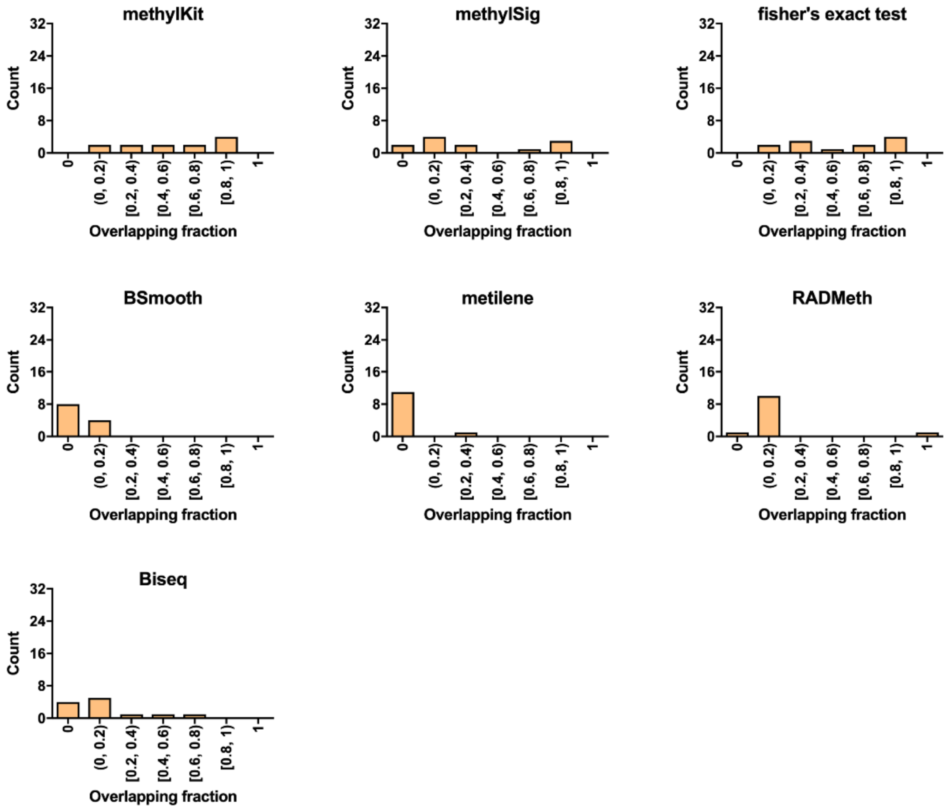

Chromosome 17

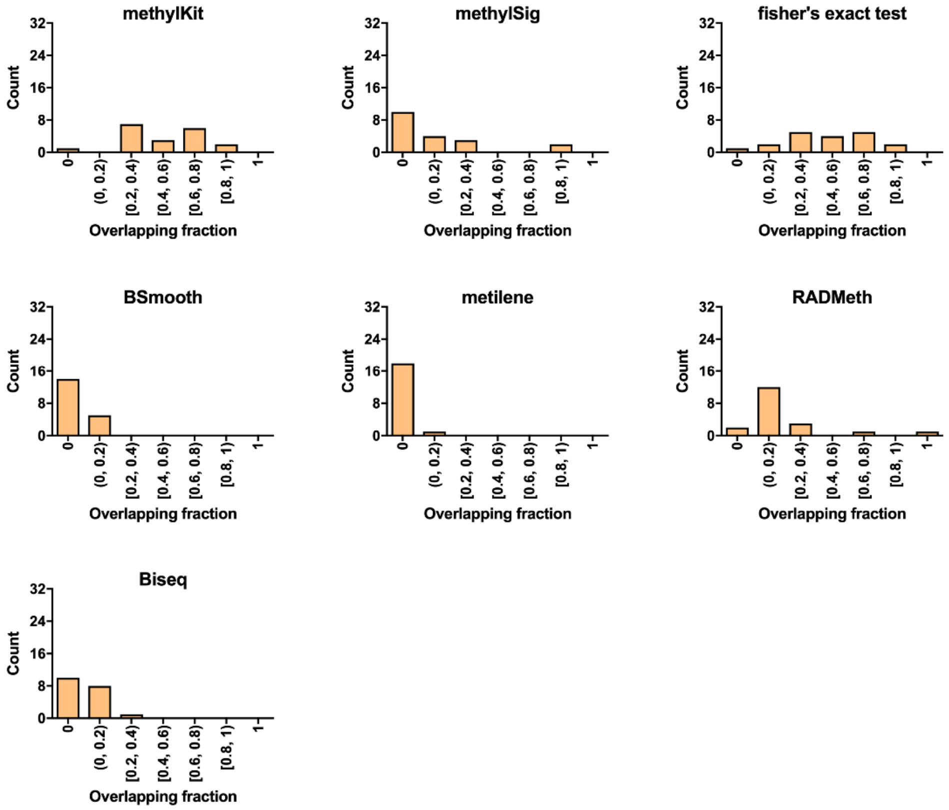

Chromosome 18

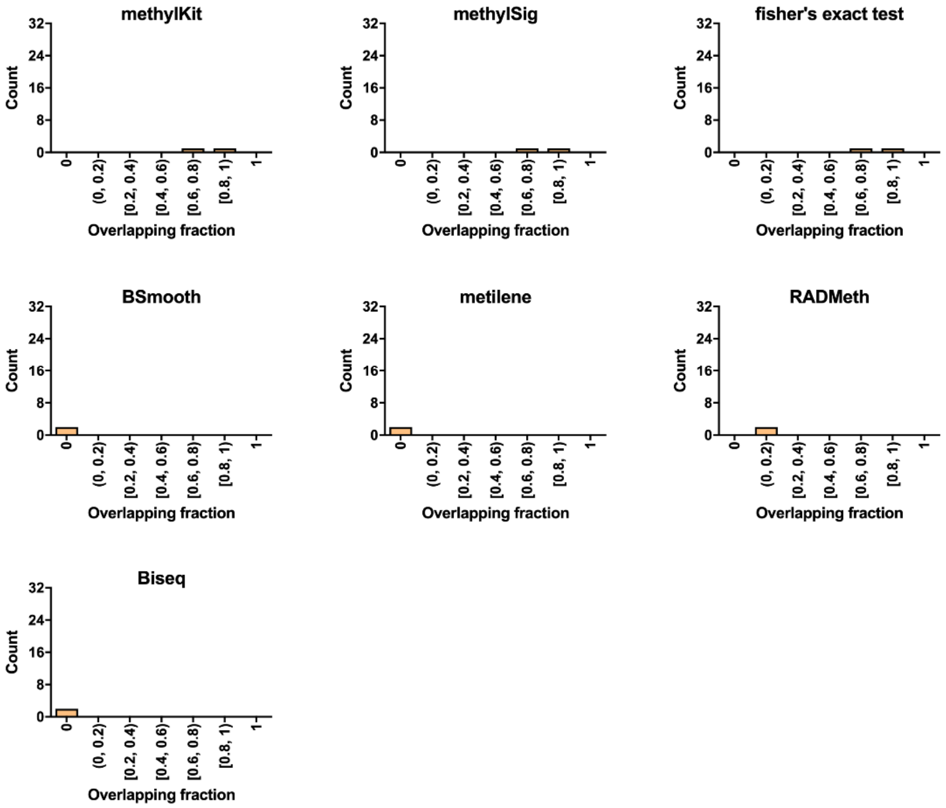

Chromosome 19

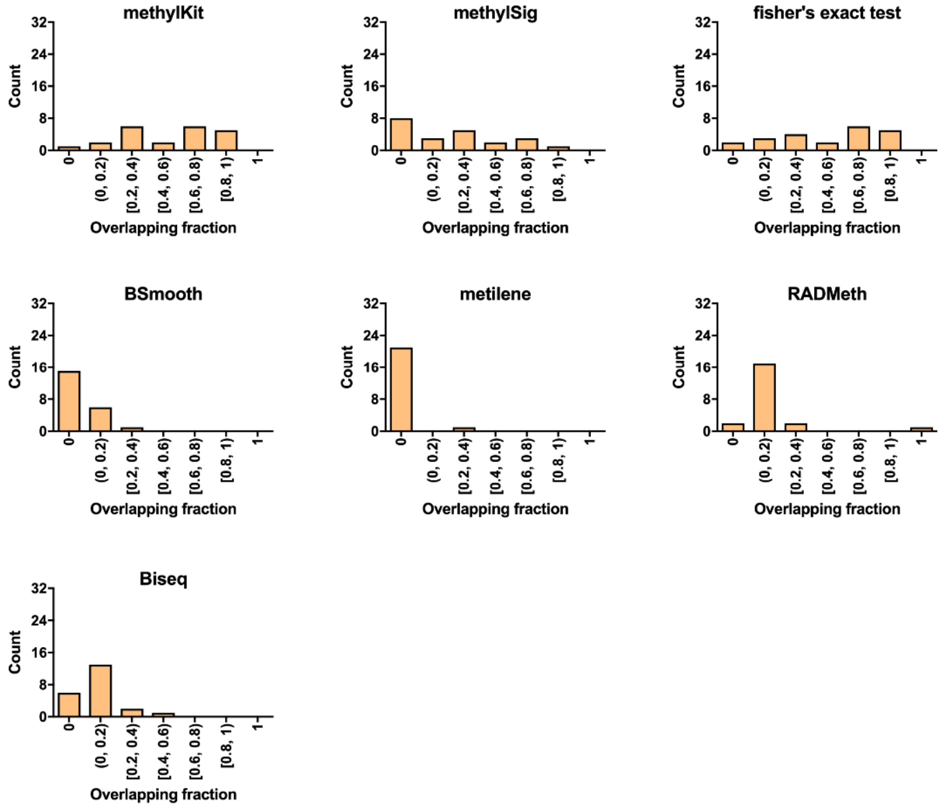

Chromosome 20

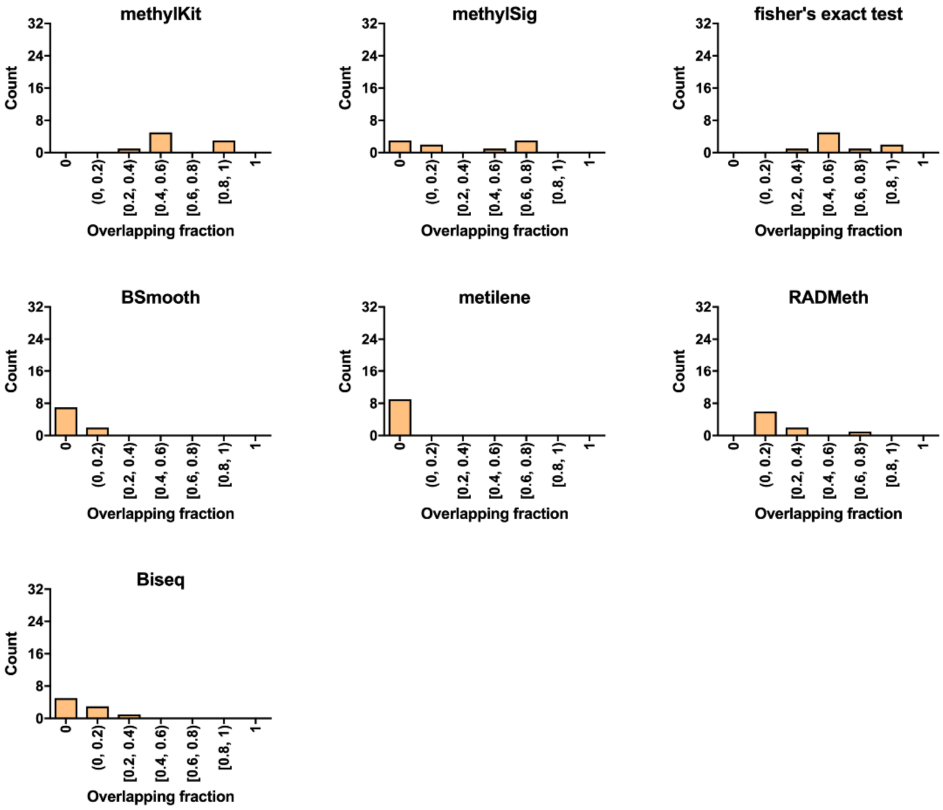

Chromosome 21

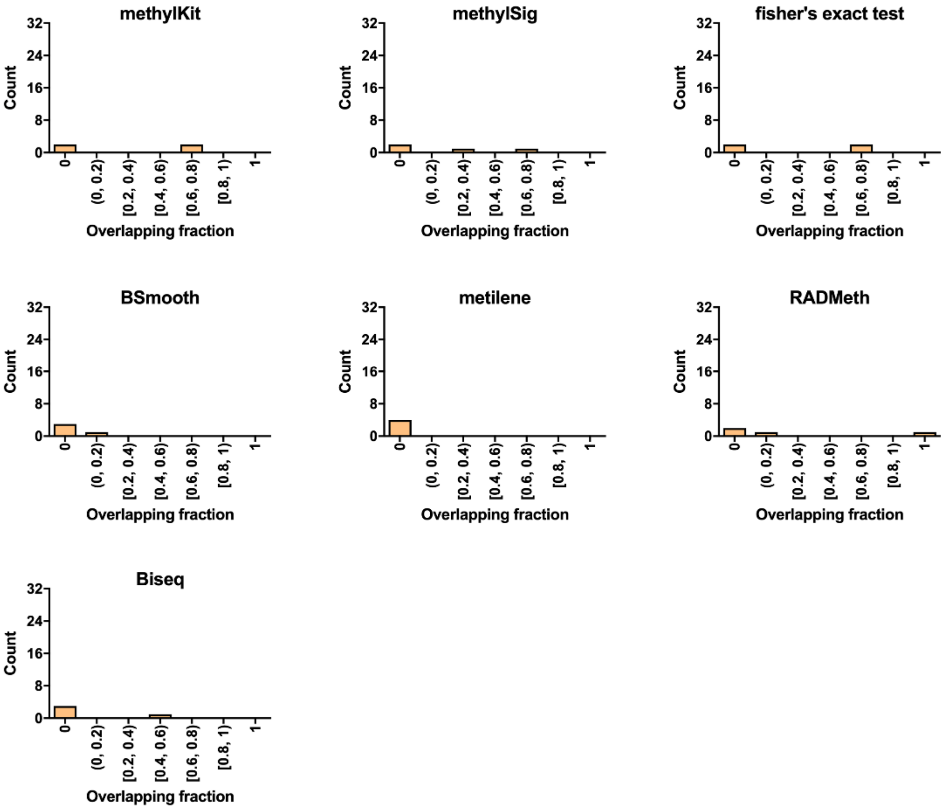

Chromosome 22

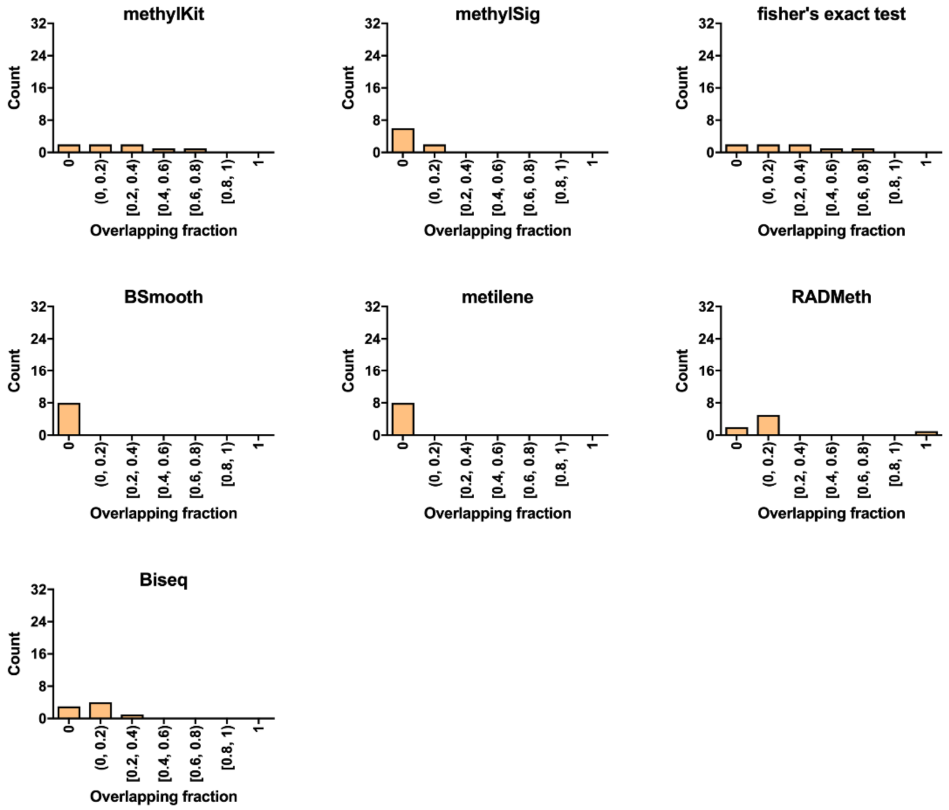

Figure S7. Overlapping fraction on IMR data between gold standard and DMRs found by each method.

Table S1. Performances for three bins of methylation difference on simulated datasets with different coverages.

0.2-0.4	methylKit		methylSig		Fisher's exact test		DSS		RADMeth		Biseq	
	TPR	FPR	TPR	FPR	TPR	FPR	TPR	FPR	TPR	FPR	TPR	FPR
5x	0.266	0.023	-	-	0.082	0.029	0.065	0.029	0.421	0.018	0.684	0.010
10x	0.963	0.001	0.716	0.009	0.920	0.002	0.935	0.002	0.967	0.001	0.607	0.012
15x	0.987	0.000	0.896	0.003	0.980	0.001	0.984	0.001	0.987	0.000	0.698	0.009
20x	0.998	0.000	0.947	0.002	0.996	0.000	0.996	0.000	0.997	0.000	0.702	0.009
25x	1.000	0.000	0.966	0.001	0.999	0.000	0.998	0.000	0.998	0.000	0.690	0.010
0.4-0.6	methylKit		methylSig		Fisher's exact test		DSS		RADMeth		Biseq	
	TPR	FPR	TPR	FPR	TPR	FPR	TPR	FPR	TPR	FPR	TPR	FPR
5x	0.857	0.013	-	-	0.600	0.036	0.737	0.024	0.964	0.003	0.658	0.031
10x	0.962	0.003	0.714	0.026	0.922	0.007	0.938	0.006	0.970	0.003	0.601	0.036
15x	0.987	0.001	0.888	0.010	0.979	0.002	0.984	0.001	0.985	0.001	0.674	0.029
20x	0.996	0.000	0.945	0.005	0.993	0.001	0.993	0.001	0.996	0.000	0.677	0.029
25x	1.000	0.000	0.969	0.003	0.999	0.000	0.999	0.000	0.997	0.000	0.666	0.030
0.6-0.8	methylKit		methylSig		Fisher's exact test		DSS		RADMeth		Biseq	

	TPR	FPR	TPR	FPR	TPR	FPR	TPR	FPR	TPR	FPR	TPR	FPR
5x	0.998	0.000	-	-	0.973	0.002	0.997	0.000	1.000	0.000	0.657	0.027
10x	0.964	0.003	0.730	0.021	0.923	0.006	0.938	0.005	0.971	0.002	0.612	0.030
15x	0.983	0.001	0.896	0.008	0.976	0.002	0.980	0.002	0.984	0.001	0.681	0.025
20x	0.996	0.000	0.948	0.004	0.994	0.000	0.995	0.000	0.996	0.000	0.687	0.024
25x	1.000	0.000	0.971	0.002	0.999	0.000	0.999	0.000	0.998	0.000	0.679	0.025

Table S2. Performances for three bins of methylation difference on simulated datasets with different number of samples.

0.2-0.4	methylKit		methylSig		Fisher's exact test		DSS		RADMeth		Biseq	
	TPR	FPR	TPR	FPR	TPR	FPR	TPR	FPR	TPR	FPR	TPR	FPR
2s	0.000	0.030	-	-	0.000	0.030	0.058	0.029	0.058	0.029	0.001	0.030
4s	0.144	0.025	0.144	0.025	0.029	0.029	0.117	0.026	0.119	0.026	0.686	0.009
6s	0.567	0.012	0.000	0.027	0.395	0.017	0.558	0.012	0.655	0.009	0.687	0.009
8s	0.805	0.006	0.268	0.021	0.727	0.008	0.786	0.006	0.879	0.003	0.696	0.009
10s	0.983	0.001	0.871	0.007	0.966	0.002	0.975	0.001	0.992	0.000	0.677	0.017
0.4-0.6	methylKit		methylSig		Fisher's exact test		DSS		RADMeth		Biseq	
	TPR	FPR	TPR	FPR	TPR	FPR	TPR	FPR	TPR	FPR	TPR	FPR
2s	0.158	0.068	-	-	0.013	0.079	0.238	0.061	0.238	0.061	0.001	0.080
4s	0.949	0.005	0.949	0.005	0.831	0.015	0.954	0.004	0.943	0.005	0.690	0.028
6s	1.000	0.000	0.593	0.035	0.997	0.000	1.000	0.000	1.000	0.000	0.689	0.026
8s	1.000	0.000	0.994	0.001	1.000	0.000	1.000	0.000	1.000	0.000	0.700	0.026
10s	1.000	0.000	1.000	0.000	1.000	0.000	1.000	0.000	0.999	0.000	0.687	0.031
0.6-0.8	methylKit		methylSig		Fisher's exact test		DSS		RADMeth		Biseq	
	TPR	FPR	TPR	FPR	TPR	FPR	TPR	FPR	TPR	FPR	TPR	FPR
2s	0.858	0.013	-	-	0.350	0.061	0.873	0.012	0.873	0.012	0.001	0.094
4s	1.000	0.000	1.000	0.000	1.000	0.000	1.000	0.000	1.000	0.000	0.687	0.028
6s	1.000	0.000	1.000	0.000	1.000	0.000	1.000	0.000	1.000	0.000	0.696	0.029
8s	1.000	0.000	1.000	0.000	1.000	0.000	1.000	0.000	1.000	0.000	0.686	0.029
10s	1.000	0.000	1.000	0.000	1.000	0.000	1.000	0.000	1.000	0.000	0.689	0.022

Table S3. 35 gold standard DMRs for region-based simulation

Chromosome	Start	End	Length
chr1	27206027	27207939	1912
chr1	27559021	27560932	1911
chr1	29363070	29364971	1901
chr1	36416010	36417877	1867
chr1	43246030	43247892	1862
chr1	47671021	47673887	2866
chr1	51212036	51214949	2913
chr1	59315253	59317916	2663
chr1	61289130	61293130	4000
chr1	62434031	62436948	2917
chr1	64710038	64712926	2888
chr1	81485084	81486985	1901
chr1	81550385	81551871	1486
chr1	112853096	112854784	1688
chr1	114376002	114379860	3858
chr1	145524012	145526968	2956
chr1	154928132	154930853	2721
chr1	157242111	157243934	1823
chr1	168895010	168896976	1966
chr1	168901041	168905941	4900
chr1	173110145	173111785	1640
chr1	176502250	176503998	1748
chr1	196139165	196140840	1675
chr1	198272020	198273957	1937
chr1	198646070	198648927	2857
chr1	209638037	209640960	2923
chr1	211148078	211151000	2922
chr1	212557054	212558957	1903
chr1	215378020	215381942	3922
chr1	219116256	219118980	2724
chr1	219122011	219131993	9982
chr1	221268146	221270967	2821
chr1	230320380	230322784	2404
chr1	230324183	230326870	2687
chr1	243045000	243046927	1927

Table S4. 32 biologically verified DMRs from Xie's data

Name	Chromosome	Start	End	Length	Number of CpGs	Overlapping Fraction					
						methylKit	fisher's exact test	DSS	metilene	RADMeth	Biseq
Gpr1/Zdbf2	chr1	63296857	63327099	30243	94	0.7347067	0.7347067	0.0926526	0.032273	0.0503935	0.043086
Mcts2/H13	chr2	152512010	152512663	654	25	1	1	1	1	1	0
Nesp	chr2	174109010	174113395	4386	73	0.9990878	0.9990878	0.5083238	0.3644242	0.2898518	0
Nespas/Gnasxl	chr2	174118404	174125287	6884	25	0.998983	0.998983	0.3019032	0.3159959	0.2128432	0
Gnas1a	chr2	174152611	174153503	893	18	0.9988789	0.9988789	0.396861	0.5067264	0.5089686	0
Peg10/Sgce	chr6	4696303	4699370	3068	41	0.6732964	0.6732964	0.2181285	0.0873818	0.2266058	0
Mest (Peg1)	chr6	30685840	30689965	4126	57	0.9990303	0.9990303	0.4431515	0.4652121	0.2894545	0
Herc3/Nap1l5	chr6	58857395	58857788	394	8	1	1	0.4122137	0	0.5368957	0
Peg3/Usp29	chr7	6680067	6685920	5854	55	0.9991457	0.9991457	0.2936955	0.1903297	0.2532035	0
Snurf/Snrpn	chr7	67148026	67150169	2144	26	0.9990667	0.9990667	0.4671022	0.2673822	0.1619225	0
Ndn	chr7	69493100	69493181	82	5	1	1	0	0	1	0
Magel2	chr7	69521307	69522167	861	15	0.9988372	0.9988372	0.1139535	0	0.2104651	0
Mkrn3	chr7	69564012	69565740	1729	13	0.9994213	0.9994213	0	0	0.103588	0
Peg12	chr7	69608471	69609019	549	18	0.9981752	0.9981752	0	0	0.4032847	0
Inpp5f	chr7	135831638	135831747	110	8	1	1	1	1	1	0
H19 promoter	chr7	149763483	149765230	1748	16	0.9988552	0.9988552	0.0549513	0	0.117344	0
H19 ICR	chr7	149765791	149767931	2141	44	0.9990654	0.9990654	0.4883178	0.8850467	0.4957944	0
Kcnq1ot1	chr7	150480736	150482006	1271	45	0.9984252	0.9984252	0.4606299	0.7204725	0.5125985	0
Cdkn1c	chr7	150645240	150647381	2142	35	0.9990659	0.9990659	0.0999533	0.1041569	0.2297992	0
Cdkn1c upstream	chr7	150649567	150649883	317	8	1	1	0	0	0.2816456	0
Rasgrf1	chr9	89771945	89780072	8128	54	0.875969	0.875969	0.2499077	0.1969977	0.0927772	0
Plagl1	chr10	12809928	12812145	2218	29	0.9986468	0.9986468	0.1695985	0.1452413	0.4379793	0
Grb10	chr11	11923325	11926800	3476	25	0.7116547	0.7116547	0.0892086	0.2152518	0.0972662	0
Zrsr1/Commd1	chr11	22871545	22874145	2601	47	0.6146154	0.6146154	0.3376923	0.4561538	0.305	0
Dlk1	chr12	110697919	110700243	2325	15	0.9987091	0.9987091	0.035284	0	0.052926	0
Dlk1-Gtl2 IG	chr12	110763965	110768609	4645	48	0.9989234	0.9989234	0.0704134	0.1057278	0.1143411	0
Gtl2	chr12	110777813	110781249	3437	40	0.9988359	0.9988359	0.1865541	0.4135623	0.3274156	0
Peg13/Trappc9	chr15	72632245	72641614	9370	133	0.9990394	0.9990394	0.4022841	0.3664212	0.2946953	0
Slc38a4	chr15	96884431	96886172	1742	29	0.9988512	0.9988512	0.3072947	0.4170017	0.3871338	0
Airn/Igf2r	chr17	12934626	12935815	1190	42	0.999159	0.999159	0.6484441	0.8158116	0.8528175	0
Igf2r	chr17	12962643	12962696	54	7	1	1	0	0	1	0
Impact	chr18	13131356	13133257	1902	64	0.9989479	0.9989479	0.5970542	0.6170437	0.526565	0

Table S5. DEGs bewteen IMR90 and H1-hESC cell

Symbol	Chromosome	Gene.start..bp.	Gene.end..bp.	Strand	logFC	logCPM	PValue
CD99	X	2691179	2741309	1	-4.137835	6.338284	0.011782217
ICA1	7	8113184	8262687	-1	4.606662	4.054853	0.023911109
COPZ2	17	48026167	48038030	-1	-8.233241	5.232882	0.001018465
PROM1	4	15963076	16084378	-1	5.353464	4.20344	0.018183048
AASS	7	122075647	122144280	-1	4.385276	6.127019	0.009569348
ST3GAL1	8	133454848	133571940	-1	-4.052515	3.969392	0.02777588
DCN	12	91140484	91183123	-1	-9.58997	6.043853	0.0002697
CLDN11	3	170418865	170860380	1	-5.302808	4.72501	0.008069191
MVP	16	29820394	29848039	1	-7.267622	7.469369	0.000188655
ATP1A2	1	160115759	160143591	1	5.176738	3.501815	0.019833567
HGF	7	81699006	81770438	-1	-7.822538	4.527403	0.003290403
CDH1	16	68737225	68835548	1	4.537262	5.060612	0.009818489
TNC	9	115019578	115118257	-1	-5.429609	6.352683	0.002091661
PREX2	8	67952118	68237030	1	5.330289	3.501177	0.019833567
DKK3	11	11963106	12009769	-1	-4.617513	7.898893	0.004880247
ATP2B4	1	203626561	203744081	1	-3.969264	5.166447	0.022798378
CDH3	16	68636189	68722616	1	5.305522	6.18891	0.002715452
GPC1	2	240435671	240468078	1	-3.367745	5.588013	0.03832954
SLC9A3R2	16	2025356	2039026	1	-3.719752	5.900726	0.02338388
ERBB3	12	56079857	56103505	1	5.538332	4.583299	0.009765631
MYLK	3	123610049	123884331	-1	-4.710407	8.282125	0.00404515
DDX3Y	Y	12904108	12920478	1	5.028864	5.522543	0.004598155
SP100	2	230415942	230544090	1	-6.305501	3.944565	0.009268927
PDZD4	X	153802166	153830565	-1	4.962907	4.631853	0.009297076
PITX1	5	135027735	135034813	-1	-6.846551	3.200153	0.032634429
GAL	11	68683779	68691175	1	4.166582	5.126128	0.015559163
ADGRL1	19	14147743	14206187	-1	5.132645	4.963159	0.005517524
TRHDE	12	72087266	72670757	1	-3.676028	3.648569	0.046557785
PVR	19	44643798	44663583	1	-3.530763	5.862965	0.031026922
LLGL2	17	75525080	75575208	1	5.180411	3.02958	0.043831579
MGLL	3	127689062	127823250	-1	-6.34016	3.428251	0.022250379
NTN4	12	95657807	95791152	-1	-6.557875	3.607998	0.016006288
ADD2	2	70607618	70768225	-1	5.552057	5.717404	0.003279591
FOSL2	2	28392448	28417312	1	-5.478314	5.623066	0.00385991

ANKRD13A	12	109999186	110039763	1	-3.725789	6.612924	0.019641408
TPD52	8	80034745	80231232	-1	4.968891	3.75577	0.038694787
NMRK2	19	3933103	3942416	1	4.844692	3.736275	0.038694787
FAP	2	162170684	162245151	-1	-6.42818	3.321975	0.025109373
NEBL	10	20779973	21174187	-1	4.581366	3.061241	0.043831579
SSH1	12	108778192	108857590	-1	-3.494702	4.952565	0.045231117
COL16A1	1	31652247	31704319	-1	-3.883315	5.687068	0.019656544
CD59	11	33698261	33736445	-1	-4.233977	5.287671	0.018308557
CD82	11	44564427	44620363	1	-5.416112	5.120401	0.004115763
PPP1R15A	19	48872392	48876057	1	-3.286301	5.611068	0.045651393
DNMT3B	20	32762385	32809356	1	4.878726	5.963321	0.006188567
CERS4	19	8206736	8262421	1	3.50213	4.933898	0.033734635
MAP3K20	2	173075435	173268010	1	-3.574256	4.85639	0.036499336
CCDC80	3	112596794	112649530	-1	-3.400196	5.449743	0.037533874
SEMA6A	5	116443616	116574934	-1	4.428192	4.704681	0.017241952
COL9A3	20	62816244	62841159	1	4.286016	4.015403	0.025740584
IL11	19	55364389	55370463	-1	-5.444877	4.663062	0.008859886
SCD	10	100347124	100364834	1	3.336148	6.975996	0.032617175
NRP1	10	33177492	33336262	-1	-4.422467	7.432281	0.007054735
PRODH	22	18912777	18936553	-1	4.499921	3.022427	0.043831579
LGALS1	22	37675608	37679806	1	-7.463919	9.164045	6.84E-05
TIMP3	22	32801701	32863043	1	-4.743275	4.724754	0.016483503
SUN2	22	38734725	38794143	-1	-3.561086	4.52374	0.040207296
MYH9	22	36281281	36388018	-1	-2.989802	7.895377	0.04869413
SALL4	20	51782331	51802520	-1	4.248784	4.140535	0.020768796
PLP2	X	49171926	49175239	1	-4.441565	4.101629	0.022261254
PIM2	X	48913182	48919024	-1	4.007948	3.653799	0.046557785
PCYT1B	X	24558087	24672677	-1	5.005882	4.270462	0.017059606
WFDC1	16	84294646	84329851	1	-5.806878	3.593654	0.016006288
CRISPLD2	16	84819984	84920768	1	-4.061453	3.857707	0.032594146
FOXF1	16	86510527	86515418	1	-6.456595	2.986074	0.043831579
SCG3	15	51681353	51721031	1	4.740979	3.886768	0.03004806
TRPA1	8	72019917	72075617	-1	-4.996616	3.018693	0.043831579
PLAT	8	42175233	42207724	-1	-6.286497	4.742443	0.00737528
ESRP1	8	94641074	94707466	1	5.284736	5.923673	0.004156955
DMPK	19	45769717	45782552	-1	-3.17551	6.888064	0.041213919
TNNT1	19	55132794	55149354	-1	4.828365	8.494437	0.003455605
DENND3	8	141117278	141195808	1	-4.239873	4.050006	0.023911109
CLEC11A	19	50723329	50725718	1	-6.447599	6.493959	0.000930791
LSR	19	35248330	35267964	1	5.0926	4.025282	0.023911109
CAV1	7	116524785	116561184	1	-4.164096	5.787022	0.012207861
CPED1	7	120988677	121297444	1	-5.695548	3.242948	0.028521587

HSPB1	7	76302544	76304295	1	-3.877787	6.842377	0.015744202
PTPRZ1	7	121873089	122062036	1	4.743868	5.746222	0.008742698
SERPINE1	7	101127089	101139266	1	-6.217326	7.305078	0.000697204
RARRES2	7	150338317	150341674	-1	4.289258	6.529484	0.010686933
SFXN3	10	101031234	101041244	1	-3.817677	3.643343	0.046557785
DKK1	10	52314296	52318042	1	-6.57975	4.064331	0.00734961
HOXB6	17	48595751	48604992	-1	-7.633935	3.852619	0.010068124
PPP1R9B	17	50133735	50150630	-1	-3.34898	7.277799	0.031301167
COL1A1	17	50183289	50201632	-1	-4.455812	9.001203	0.005822835
MAP2K6	17	69414698	69543331	1	3.624801	4.920778	0.033734635
CCND1	11	69641087	69654474	1	-3.732185	7.48565	0.018949899
WNT5B	12	1529891	1647243	1	-7.234029	5.193972	0.00112286
SCNN1A	12	6346843	6377730	-1	4.762976	4.023039	0.025740584
LTBR	12	6375045	6391571	1	-4.608792	3.918929	0.03004806
NANOG	12	7787794	7799141	1	5.37023	4.488417	0.011391147
PHC1	12	8913896	8941467	1	3.947948	6.449262	0.015150404
COL12A1	6	75084326	75206051	-1	-6.063281	6.302992	0.001268542
COL9A1	6	70215061	70303083	-1	4.787176	3.404998	0.022250379
HMGCS1	5	43289395	43313512	-1	3.688226	6.495385	0.023332889
SPARC	5	151661096	151687165	-1	-3.504488	10.75009	0.022833915
STC2	5	173314713	173329503	-1	-3.128795	7.995688	0.041459163
CLIP4	2	29097705	29189643	1	-6.933229	3.671442	0.014478713
FN1	2	215360440	215436172	-1	-3.800085	9.560391	0.015047242
IGFBP2	2	216632828	216664436	1	4.115622	6.713298	0.011948077
IGFBP5	2	216672105	216695525	-1	-7.708455	11.13995	3.50E-05
IL1R1	2	102064544	102179874	1	-5.513211	3.020473	0.043831579
SLC1A4	2	64988477	65023865	1	-6.347687	4.192485	0.005950201
RND3	2	150468195	150539011	-1	-3.153662	5.593155	0.047851135
EPAS1	2	46293667	46386703	1	-7.451969	6.58979	0.000368288
PRRX1	1	170662728	170739419	1	-5.591806	5.227305	0.003485289
WLS	1	68098473	68233120	-1	-5.469333	4.723084	0.007711136
GBP3	1	89006666	89022894	-1	-9.114768	5.202659	0.001086434
GBP1	1	89052319	89065360	-1	-8.055472	7.055166	0.000171304
F3	1	94529225	94541800	-1	-5.931476	6.488968	0.001653629
TNNT2	1	201359008	201377762	-1	4.056614	3.878365	0.032594146
SGIP1	1	66533383	66748299	1	-5.22962	4.590837	0.009765631
CTGF	6	131948176	131951373	-1	-3.649741	5.022089	0.029032968
PPL	16	4882507	4960741	-1	4.575379	3.889215	0.03004806
CCDC92	12	123918660	123972831	-1	-5.894054	3.315437	0.025109373
LTBP2	14	74498170	74612378	-1	-4.838906	3.447694	0.022250379
EPCAM	2	47345158	47387601	1	4.992151	7.147713	0.003169642

HOXB8	17	48611377	48614939	-1	-6.421962	3.378884	0.022250379
HOXB3	17	48548870	48604912	-1	-5.963765	4.064773	0.00734961
CD274	9	5450503	5470566	1	-6.333267	3.11562	0.037646741
TGFBI	5	136028895	136063818	1	-5.506931	6.461494	0.00272036
PLS1	3	142596387	142713664	1	3.522966	4.103358	0.047009089
CRISPLD1	8	74984515	75034558	1	4.333908	3.836786	0.035458532
AKAP1	17	57085092	57121349	1	3.528059	5.877365	0.030421178
TBX2	17	61399896	61409466	1	-6.604396	3.098776	0.037646741
DPPA4	3	109326141	109337572	-1	5.582419	5.643321	0.003768469
FKBP9	7	32957404	33006931	1	-4.042586	6.161032	0.015540101
PLAU	10	73909177	73917497	1	-5.51535	6.473384	0.002684391
CDKN2C	1	50960745	50974633	1	-6.406156	3.172256	0.032634429
OPTN	10	13099449	13138308	1	-3.857095	5.135825	0.023564679
CDKN1A	6	36676460	36687339	1	-4.596486	6.072235	0.007537238
RAB17	2	237574322	237601614	-1	4.328791	4.023468	0.025740584
AHNAK	11	62433542	62556235	-1	-6.66699	7.065181	0.000365421
MCF2L	13	112894378	113099739	1	4.692738	3.658437	0.046557785
AIF1L	9	131096476	131123152	1	5.649468	5.588061	0.004052512
CTAG2	X	154651972	154653579	-1	-6.672315	3.52092	0.017774094
TNFRSF19	13	23570370	23676104	1	-6.986946	5.582431	0.001878731
KDR	4	55078477	55125589	-1	5.069153	4.783052	0.00737528
LIF	22	30240447	30246851	-1	-4.034488	4.19414	0.038506916
CPA4	7	130293134	130324180	1	-6.816947	4.231242	0.005567924
PODXL	7	131500262	131558217	-1	4.966773	7.247277	0.003368718
FLNC	7	128830377	128859274	1	-4.527114	3.874603	0.032594146
LRRC17	7	102912991	102944949	1	-5.05984	4.955989	0.011380843
CGNL1	15	57375967	57550727	1	4.808201	3.829668	0.032594146
PALLD	4	168497066	168928457	1	-3.739503	6.31681	0.022218174
CD68	17	7579467	7582113	1	-6.11965	3.080026	0.037646741
AP1M2	19	10572671	10587315	-1	4.698582	4.416057	0.013430763
RPS4Y1	Y	2841486	2932000	1	5.454735	5.397665	0.005554949
TNNI3	19	55151767	55157773	-1	4.526085	4.589314	0.020829491
GDPD2	X	70423031	70433390	1	4.737688	3.11086	0.037646741
CNN1	19	11538717	11550323	1	-3.036684	7.785831	0.046492388
ZSCAN10	16	3088890	3099317	-1	5.161267	4.722429	0.008069191
APOE	19	44905754	44909393	1	4.971883	9.212218	0.002651911
COL5A1	9	134641774	134844843	1	-3.541079	5.116945	0.035327229
IDO1	8	39902275	39928444	1	5.505172	3.671631	0.014478713
LIN28A	1	26410778	26429722	1	5.083036	7.640851	0.002668799
RIN2	20	19886521	20002457	1	-6.946616	5.267396	0.000986735
SYT4	18	43267878	43277650	-1	4.886794	3.100738	0.037646741
BEX2	X	103309346	103311046	-1	5.315786	5.306245	0.00642134
BEX1	X	103062651	103064240	-1	4.821055	6.943653	0.004366899

NTS	12	85874295	85882992	1	4.858564	3.871963	0.032594146
MICAL2	11	12094008	12263789	1	-6.256726	7.098605	0.00061708
VRTN	14	74303069	74360008	1	5.331541	6.001031	0.00370955
LOXL2	8	23297189	23425328	-1	-3.754003	6.468398	0.0209158
IL6ST	5	55935095	55994993	-1	-4.675809	5.055361	0.009818489
FST	5	53480409	53487134	1	-4.33315	6.349172	0.008895283
EMP1	12	13196716	13219939	1	-6.252378	5.728761	0.001519009
STK26	X	132023265	132075943	1	5.262438	4.308363	0.015091094
HOOK1	1	59814786	59876378	1	6.761813	3.994192	0.007920696
PDGFRA	4	54229097	54298247	1	-5.902846	4.495933	0.011391147
COL4A2	13	110305812	110513027	1	-3.331158	9.725228	0.029706087
ANXA1	9	73151757	73170393	1	-4.499014	6.26411	0.007650055
BICDL1	12	119989869	120094494	1	5.043466	3.983179	0.025740584
NT5E	6	85449584	85495791	1	-6.781037	5.773346	0.001390151
MAP7	6	136342281	136550819	-1	4.517437	4.249209	0.018183048
CNMD	13	52703264	52739812	-1	5.031909	5.572403	0.007342539
LMO7	13	75620434	75859870	1	-3.141872	6.739726	0.043032098
GALNT5	2	157257598	157314211	1	-7.648401	4.627958	0.002826005
FAM129B	9	127505339	127578989	-1	-3.179965	7.540053	0.038248153
CTSV	9	97029679	97039643	-1	4.443257	7.370464	0.006735423
IER3	6	30743199	30744554	-1	-3.586619	7.197244	0.022879852
SYTL2	11	85694224	85811159	-1	-3.604052	5.706535	0.025437989
THBS1	15	39581079	39599466	1	-6.138902	7.784798	0.000650055
EMILIN1	2	27078567	27086408	1	-5.572549	6.269159	0.002392965
MYOF	10	93306429	93482317	-1	-6.223408	5.545012	0.002025713
STAMBPL1	10	88879734	88975153	1	-4.316623	4.644947	0.018915262
SSFA2	2	181891833	181930738	1	-4.678776	6.316206	0.006993011
ARHGAP24	4	85475114	86002670	1	-5.539746	3.828841	0.010969165
FGF5	4	80266599	80336680	1	-7.458837	4.198811	0.005950201
PDE5A	4	119494395	119628991	-1	-3.718428	5.64782	0.027234414
11-Sep	4	76949703	77040384	1	-3.057504	10.12133	0.043175447
GLIPR1	12	75480680	75503853	1	-6.17648	3.296357	0.028521587
SLC27A2	15	50182196	50236395	1	5.267371	2.990811	0.043831579
FGF7	15	49423096	49488775	1	-8.827761	5.036854	0.001434211
KIFC3	16	57758217	57863053	-1	-4.509077	5.143403	0.014550253
CDH11	16	64943753	65126112	-1	-4.004094	4.542513	0.02303162
TMC6	17	78110458	78132407	-1	5.782599	4.177587	0.005950201
IGFBP4	17	40443461	40457731	1	-4.150997	7.146683	0.011106963
COL6A1	21	45981737	46005050	1	-3.587109	7.550809	0.021991753
COL6A2	21	46098097	46132849	1	-3.662867	8.061868	0.018887201
EMP3	19	48321509	48330553	1	-3.64281	5.787979	0.028032021

CGN	1	151510510	151538692	1	4.53289	4.401687	0.013430763
VASH2	1	212950520	212992037	1	4.864949	4.160197	0.019414798
DUSP10	1	221701424	221742176	-1	-4.87228	3.09116	0.037646741
ACTA1	1	229431245	229434098	-1	4.862596	3.252514	0.028521587
MEIS1	2	66433452	66573869	1	-8.095229	5.921706	0.000332225
COL8A1	3	99638475	99799226	1	-4.70671	3.017805	0.043831579
SNCA	4	89724099	89838315	-1	4.078481	4.615903	0.020829491
CAMK2D	4	113451032	113761927	-1	-3.388179	4.460561	0.047063422
SFRP2	4	153780592	153789120	-1	5.375541	4.560077	0.010268534
IQGAP2	5	76403249	76708132	1	5.01668	5.383624	0.00571431
MEGF10	5	127290831	127465737	1	4.26003	4.812272	0.015102785
TRIM4	7	99876958	99919600	-1	-4.107626	4.745491	0.027635661
EPHA1	7	143390289	143408892	-1	4.579802	4.451015	0.0126969
DENND2A	7	140518420	140673993	-1	-3.486161	6.891214	0.027361324
GPC3	X	133535745	133985895	-1	4.474824	4.018104	0.025740584
TRIM55	8	66126896	66175487	1	-7.272121	3.888338	0.010068124
TERF1	8	73008864	73048122	1	4.585883	9.732824	0.004622056
NR6A1	9	124517275	124771310	-1	4.446585	4.259104	0.017059606
ZEB1	10	31318495	31529814	1	-8.344774	6.43378	0.000143256
PAMR1	11	35431823	35530300	-1	-4.95725	4.242053	0.017059606
ST14	11	130159562	130210376	1	4.555513	3.205242	0.032634429
TAGLN	11	117199321	117204782	1	-3.332247	9.906075	0.02948351
ARID5B	10	61901300	62096944	1	-5.5918	4.415943	0.0126969
LYPD1	2	132644853	132671579	-1	-6.25901	5.403391	0.002579247
VEGFC	4	176683538	176792727	-1	-4.704686	3.654295	0.046557785
PRSS23	11	86791059	86952910	1	-4.256342	4.010482	0.025740584
CRIM1	2	36355926	36551135	1	-3.836134	4.986811	0.0301165
EPS8	12	15620158	15882329	-1	-3.81105	4.473652	0.042325029
CCDC50	3	191329077	191398670	1	-3.209914	6.452381	0.038446327
MBNL1	3	152243828	152465780	1	-3.624545	4.565398	0.038237087
DAB2	5	39371675	39462300	-1	-4.350634	3.656612	0.046557785
AK5	1	77282051	77559969	1	-4.693429	6.891743	0.005989692
RHOC	1	112701106	112707434	-1	-3.729466	7.844523	0.018317363
SLC16A1	1	112911847	112957013	-1	3.056428	7.53919	0.045722254
FMN2	1	240014348	240475189	1	-7.65532	4.510632	0.003469421
MAP3K7CL	21	29077471	29175889	1	-7.596881	3.820625	0.010969165
MMP14	14	22836557	22849027	1	-3.102361	7.075613	0.042919073
CREB3L1	11	46277661	46321422	1	-4.354912	6.235128	0.010640542
KRTCAP3	2	27442366	27446481	1	5.328934	3.446251	0.022250379
PPP1R9A	7	94907202	95296415	1	5.156597	3.107503	0.037646741
TAGLN2	1	159918107	159925732	-1	-3.533697	7.991631	0.023591697
F11R	1	160995211	161021348	-1	4.980634	3.882349	0.03004806
CSRP1	1	201483530	201509456	-1	-4.83671	6.369562	0.004697745

C1R	12	7080209	7092607	-1	-3.849602	4.086363	0.047009089
SPON2	4	1166932	1208962	-1	-6.298434	5.358292	0.002731125
LMNA	1	156082573	156140089	1	-4.957865	8.712169	0.002892899
DMKN	19	35497220	35513658	-1	4.901432	5.897087	0.006815405
ITGA5	12	54395261	54419460	-1	-3.603469	4.501804	0.042325029
SYNC	1	32680360	32703596	-1	-6.805605	4.463401	0.003662633
CAMK2N1	1	20482391	20486220	-1	-4.792465	4.682144	0.018051047
ALPL	1	21509372	21578412	1	4.689592	4.233081	0.018183048
MXRA8	1	1352689	1361777	-1	-5.157594	6.700149	0.002752764
NEXN	1	77888513	77943895	1	-5.222948	6.994422	0.002358272
GBP2	1	89106132	89150456	-1	-8.000504	5.618612	0.00055131
DDR2	1	162631373	162787400	1	-5.67354	4.329082	0.015091094
CAPN2	1	223701593	223776018	1	-6.163213	8.022644	0.000587385
ACTG2	2	73892314	73919865	1	-5.534496	6.073387	0.003269547
VSNL1	2	17539126	17657018	1	4.781109	4.976361	0.010958781
COL6A3	2	237324003	237414375	-1	-7.234517	7.061432	0.000365421
LMOD1	1	201896452	201946588	-1	-5.679907	5.701146	0.003354546
IGFBP7	4	57030773	57110385	-1	-6.108407	5.505151	0.002189626
IFI16	1	158999968	159055155	1	-5.858805	4.176932	0.005950201
CADPS	3	62398346	62875389	-1	4.508191	3.090529	0.043831579
PTX3	3	157436789	157443628	1	-5.152973	3.981343	0.025740584
CXCL5	4	73995642	73998779	-1	5.046878	3.488281	0.019833567
CAMKV	3	49857988	49870222	-1	4.718456	4.564975	0.010268534
FAM160A1	4	151409216	151663632	1	5.004512	3.052523	0.043831579
SCGB3A2	5	147870682	147882191	1	5.041375	3.996895	0.025740584
GALNT10	5	154190730	154420984	1	-3.739216	5.228357	0.029124289
COL1A2	7	94394561	94431232	1	-4.876143	8.85877	0.003134878
CTSB	8	11842524	11869448	-1	-4.866536	6.182228	0.006267226
DLC1	8	13083361	13604610	-1	-5.016689	3.669762	0.04236799
TNFRSF11B	8	118923557	118952200	-1	-5.15719	6.025861	0.003575632
CTHRC1	8	103371515	103382997	1	-6.587781	7.037116	0.000680126
STRBP	9	123109500	123268576	-1	3.169989	6.488231	0.041332538
FAT3	11	92352096	92896470	1	4.258745	4.244605	0.036158132
SLC7A3	X	70925582	70931125	-1	5.268904	5.113219	0.008839926
JCAD	10	30012800	30115494	-1	-4.879897	4.303609	0.016032432
SALL2	14	21521081	21537216	-1	3.832382	5.449881	0.020636304
FBN1	15	48408306	48645849	-1	-5.00645	5.596035	0.00716402
CRABP1	15	78340324	78348230	1	5.066195	4.372543	0.014226513
B2M	15	44711477	44718877	1	-4.204907	6.522172	0.010822995
NNMT	11	114257787	114313285	1	-6.906154	5.181633	0.00112286
GREM1	15	32717974	32745107	1	-8.257422	8.291865	4.90E-05
MAP1A	15	43510958	43531620	1	-5.326189	6.011756	0.003641715

CERCAM	9	128411751	128437351	1	-4.241174	4.744401	0.016483503
TPM4	19	16067021	16103005	1	-3.149976	13.28489	0.037430901
AXL	19	41219203	41261766	1	-3.9771	6.814856	0.014098436
CDC42EP5	19	54465026	54473264	-1	-4.450701	4.238233	0.018183048
SPINT2	19	38244035	38292614	1	4.869953	5.115303	0.008839926
IGFBP6	12	53097436	53102345	1	-4.013947	4.566903	0.02189121
HRASLS5	11	63461404	63491194	-1	4.99788	3.426785	0.022250379
SCARA3	8	27633868	27676776	1	-3.98876	3.688496	0.046557785
KIF5C	2	148875250	149026759	1	5.059666	5.400063	0.005554949
FILIP1L	3	99830141	100114513	-1	-7.389728	4.608461	0.002969807
BDKRB2	14	96204679	96244166	1	-7.821526	4.016256	0.007920696
CAVIN2	2	191834302	191847255	-1	-7.703093	3.912629	0.009268927
ADAM9	8	38996869	39105144	1	-4.136825	7.018345	0.011833882
FAM84B	8	126552442	126558393	-1	4.543417	3.862493	0.032594146
IL7R	5	35852695	35879603	1	-5.586101	3.510148	0.019833567
CHRNA5	15	78565520	78595269	1	3.485021	4.485734	0.04460492
ZFPM2	8	104590733	105804532	1	-6.385266	4.521741	0.003469421
KRT8	12	52897187	52949954	-1	3.465257	8.717141	0.024963025
SGCD	5	155870344	156767788	1	-6.77694	3.233709	0.028521587
KRT19	17	41523617	41528308	-1	4.550821	5.801705	0.008022553
GAP43	3	115623324	115721490	1	4.587031	3.970244	0.02777588
EFEMP2	11	65866441	65873592	-1	-3.968307	4.40714	0.028550021
SLFN11	17	35350305	35373701	-1	-8.214574	5.215217	0.001051668
MIR4435-2HG	2	111196350	111495100	-1	-4.283736	5.548334	0.012138775
LRRN3	7	111091006	111125454	1	-7.347117	5.141287	0.001201116
ABLIM3	5	149141483	149260542	1	-6.686389	3.244468	0.028521587
ZNF483	9	111525159	111577844	1	4.552293	3.017952	0.043831579
EHBP1L1	11	65576038	65592650	1	-3.884744	4.623054	0.034688451
NABP1	2	191678068	191696659	1	-4.848483	6.744075	0.004697137
FGFBP3	10	91906589	91909483	-1	4.519204	4.339173	0.015091094
CD248	11	66314487	66317044	-1	-5.510111	3.448343	0.022250379
FOSL1	11	65892049	65900573	-1	-4.93803	3.004298	0.043831579
LRRN1	3	3799437	3847703	1	4.903764	4.897883	0.012297254
NUPR1	16	28532708	28539174	-1	-9.721499	6.944411	6.11E-05
JAKMIP2	5	147585439	147782848	-1	5.939683	3.803918	0.011989555
FOXL1	16	86576368	86582160	1	-7.222918	3.919841	0.009268927
BDNF	11	27654893	27722058	-1	-3.811189	3.764156	0.038694787
CAVIN1	17	42402452	42423517	-1	-5.097267	7.955567	0.002624862
JUN	1	58780788	58784327	-1	-3.376708	5.013826	0.042035357
TRIML2	4	188091273	188109603	-1	4.506111	3.769669	0.038694787
ZFP42	4	187995771	188005050	1	4.929663	3.976269	0.025740584
GREM2	1	240489573	240612149	-1	-6.537968	4.328708	0.004610543

CHRM2	7	136868669	137020255	1	-8.25285	4.401912	0.004097829
SOX2	3	181711924	181714436	1	5.091964	6.693408	0.003816748
TNRC18	7	5306790	5425414	-1	-3.494117	4.943397	0.045231117
MXRA7	17	76672551	76711016	-1	-3.543174	5.77506	0.028627741
ANXA2	15	60347134	60402883	-1	-3.129745	9.299482	0.03946907
LCK	1	32251239	32286165	1	5.152919	3.384286	0.025109373
SLC8A1	2	40097270	40611053	-1	-4.139135	4.99728	0.01925934
GAS6	13	113820549	113864067	-1	-5.625862	6.933921	0.001868102
FAM46C	1	117605934	117628372	1	-7.314957	4.381524	0.004343433
SMTN	22	31064105	31104757	1	-3.682948	6.343152	0.021587963
COLEC10	8	118995452	119106582	1	-6.46968	3.694047	0.013150652
TMEM173	5	139475534	139482935	-1	-5.038132	3.547253	0.017774094
NELL2	12	44508275	44921848	-1	5.506186	4.539922	0.010809124
CLDN6	16	3014712	3020071	-1	4.883367	5.533402	0.007917714
TCEAL2	X	102125688	102127711	1	4.719233	4.184367	0.019414798
SP140L	2	230327184	230403732	1	-4.139485	3.983218	0.02777588
NTF3	12	5432112	5521536	1	-4.814977	4.574803	0.010268534
SYN3	22	32512552	33058372	-1	4.688874	3.07424	0.043831579
LEMD1	1	205381378	205457091	-1	4.747019	3.918823	0.03004806
NAP1L3	X	93670930	93673568	-1	4.334632	4.590512	0.020829491
TRDN	6	123216339	123637093	-1	5.058608	8.769818	0.002425548
NAP1L2	X	73212299	73214848	-1	5.028425	4.314942	0.015091094
MITF	3	69739435	69968337	1	-4.427002	4.243989	0.018183048
MT1X	16	56682424	56684196	1	3.796734	4.464527	0.04460492
PALM3	19	14053365	14059159	-1	4.767092	3.179019	0.032634429
C15orf52	15	40331452	40340967	-1	-7.315539	4.203939	0.005567924
MMP1	11	102789920	102798160	-1	-4.579287	4.044285	0.023911109
DAPK1	9	87497228	87708633	1	6.052842	4.797776	0.006763324
PARVA	11	12377185	12530801	1	-4.011959	5.11398	0.024368147
FAM114A1	4	38867677	38945739	1	-4.230082	7.534415	0.008782758
OCLN	5	69492292	69558104	1	4.424329	5.499096	0.013106122
C4orf22	4	80335720	80963756	1	-6.612935	4.289915	0.00490167
S100A6	1	153534599	153536244	-1	-6.759787	8.884177	0.000187115
LPAR1	9	110873263	111038458	-1	-6.877394	5.248244	0.001018465
PEG3	19	56810083	56840728	-1	4.609489	3.125081	0.037646741
TPM2	9	35681992	35691020	-1	-3.696037	9.960598	0.017297437
ITGBL1	13	101452593	101720856	1	-4.94236	3.316184	0.028521587
EIF1AY	Y	20575725	20593154	1	5.614182	4.197914	0.018183048
FAM169A	5	74777574	74866951	-1	5.155093	3.531017	0.017774094
ALPK2	18	58481247	58628957	-1	-5.701524	3.540395	0.017774094
TGM2	20	38127387	38166578	-1	-6.214817	4.542441	0.003290403
RNA5S12	1	228634871	228634989	-1	6.848709	3.732488	0.013150652

Y_RNA	14	20679411	20679512	1	-4.512912	4.247046	0.018183048
RNU6-37P	1	10298966	10299072	1	7.448858	3.693717	0.013150652
RNA5S17	1	228646040	228646158	-1	-9.070404	5.16068	0.00116105
RNA5S4	1	228616991	228617109	-1	6.848709	3.732488	0.013150652
Y_RNA	4	157768013	157768113	-1	-7.058669	3.369782	0.022250379
SNORA33	6	132817219	132817348	1	6.981429	3.307144	0.025109373
RNA5S6	1	228621447	228621565	-1	-9.087466	5.176816	0.00112286
SNORA74A	5	139278781	139278978	1	3.401966	4.924591	0.046959633
RNA5S14	1	228639337	228639455	-1	6.848709	3.732488	0.013150652
RNA5S2	1	228612509	228612627	-1	6.848709	3.732488	0.013150652
snoU2_19	X	20136306	20136385	-1	9.805406	5.863846	0.000360122
Y_RNA	7	10901978	10902074	1	-6.771469	3.141337	0.032634429
SNORD58C	18	49489245	49489308	-1	8.239929	4.390013	0.004097829
SNORD34	19	49490904	49490974	1	8.032389	4.203076	0.005567924
COL15A1	9	98943179	99070792	1	-6.393765	3.472065	0.019833567
LAYN	11	111540280	111561745	1	-3.670286	4.258512	0.036158132
CARD16	11	105041326	105101431	-1	-7.68359	3.895667	0.009268927
ARHGEF34P	7	144272445	144286966	-1	4.309081	3.704844	0.04236799
DPF3	14	72619296	72894116	-1	-6.189821	5.548117	0.002025713
ITPRIPL2	16	19113932	19121629	1	-4.459196	3.818109	0.035458532
RP9P	7	32916815	32943176	-1	-5.591672	3.121478	0.037646741
TRIM71	3	32818018	32897826	1	5.043005	4.655685	0.008859886
RNVU1-7	1	148038753	148038916	-1	3.340316	8.007543	0.029838699
SNORD116-18	15	25085385	25085476	1	4.163355	10.03608	0.008741023
SNORD116-9	15	25073107	25073201	1	4.02876	4.116852	0.043901897
SNORD101	6	132815307	132815379	1	7.782047	3.981377	0.008557121
RNU6-4P	3	181231737	181231843	1	-5.876832	5.749551	0.003136672
SNORD116-3	15	25056860	25056954	1	4.02876	4.116852	0.043901897
Y_RNA	1	153785720	153785821	-1	-6.555486	2.975964	0.043831579
RNU6-3P	X	141118030	141118136	-1	-5.849568	5.723697	0.003279591
SNORD116-1	15	25051477	25051571	1	7.081178	3.387786	0.022250379
SNORD116-13	15	25079058	25079149	1	9.571491	5.638404	0.000525145
SNORD116-15	15	25081287	25081378	1	7.478302	3.718764	0.013150652
RNU6-2	19	1021522	1021628	1	10.53868	6.577751	0.000111581
SNORD38B	1	44778390	44778456	1	-8.972545	5.068334	0.001333839
RNU6-9	19	893484	893590	1	-5.839247	5.713916	0.003279591
MIR302A	4	112648183	112648251	-1	-6.693379	3.080879	0.037646741
MIR320A	8	22244962	22245043	-1	6.835353	3.191033	0.032634429
MT-TI	MT	4263	4331	1	7.398409	3.650982	0.014478713
MT-TA	MT	5587	5655	-1	7.980747	4.156986	0.006370969
MT-TC	MT	5761	5826	-1	-9.173275	5.258125	0.000986735
MT-TK	MT	8295	8364	1	-6.64118	3.040882	0.043831579
MT-TR	MT	10405	10469	1	6.636118	3.036698	0.043831579

MT-TL2	MT	12266	12336	1	7.296303	3.565202	0.016006288
SNORD12	20	49280683	49280772	1	7.955844	4.134826	0.006370969
SNORD69	3	52692736	52692812	1	7.607687	3.82971	0.010969165
ARHGEF35	7	144186083	144195655	-1	3.742723	4.133486	0.043901897
LBH	2	30231531	30323730	1	-4.811106	7.479986	0.004110922
MEG3	14	100779410	100861031	1	-4.01128	3.807804	0.035458532
NPIPA8	16	18317942	18336736	-1	-4.697764	5.987282	0.005960702
ARHGEF28	5	73626158	73941993	1	-6.390463	4.901409	0.005738889
TSTD1	1	161037631	161038990	-1	5.004161	3.21624	0.032634429
SNORD100	6	132816802	132816877	1	7.649702	3.866037	0.010068124
SNORD111B	16	70529509	70529588	1	-7.777523	3.97763	0.008557121
CYTOR	2	87455368	87606805	1	-3.280195	5.490999	0.045587488
SNORD12B	20	49280319	49280409	1	7.179264	3.468102	0.019833567
SNORD63B	5	138558970	138559039	-1	-7.633935	3.852619	0.010068124
CKMT1A	15	43692886	43699222	1	4.208479	3.765389	0.038694787
HLA-DPB2	6	33112451	33129084	1	5.453455	3.705437	0.013150652
SVIL-AS1	10	29409402	29487745	1	-7.570425	3.797851	0.010969165
AC079150.2	2	153208086	153208163	-1	7.868599	4.057525	0.00734961
FOXD3-AS1	1	63320884	63324441	-1	5.325273	5.284788	0.006617473
AL079301.1	22	45000565	45003619	-1	4.821243	3.265414	0.028521587
ZNF826P	19	20340269	20424969	-1	5.189965	3.888254	0.03004806
LNCPRESS1	7	101299613	101301270	1	5.249708	5.657918	0.003680082
ERVH48-1	21	42916803	42925646	-1	5.118996	3.77235	0.035458532
CCDC144NL-AS1	17	20868433	21002276	1	4.102737	4.604901	0.020829491
HOXB-AS3	17	48549630	48606414	1	-7.083543	3.389995	0.022250379
AL121761.1	20	19757708	19809675	1	-5.460405	5.420111	0.005401841
MAGI2-AS3	7	79452877	79471208	1	-4.813586	3.44703	0.022250379
LINC00458	13	54115783	54132866	-1	5.626144	5.852164	0.002645609
C12orf75	12	105235290	105396097	1	-4.227172	7.487637	0.009422096
MSC-AS1	8	71828167	72118393	1	-4.615222	3.984114	0.02777588
AL117378.1	6	131901963	131920565	1	5.319575	3.594341	0.016006288
SNORD126	14	20326450	20326526	-1	-7.313387	3.579749	0.016006288
Y_RNA	X	41316488	41316589	1	-7.169379	3.46024	0.019833567
SNORD2	3	186784796	186784864	1	3.175962	5.484471	0.045587488
SNORD13	8	33513475	33513578	1	8.286908	4.432689	0.003871538
L1TD1	1	62194831	62212328	1	5.135333	6.997476	0.003111921
HIST2H2BB	1	143875171	143904650	-1	-4.793292	5.285227	0.006822245
INMT	7	30697985	30757602	1	-4.615941	3.173094	0.037646741
AKAP2	9	110048598	110172512	1	-3.230696	5.499049	0.04443693
AC005062.1	7	19918981	20140453	-1	5.191195	7.464274	0.002370029
NR2F2-AS1	15	96110040	96327361	-1	-4.922323	3.610999	0.046557785
FMN1	15	32765545	33194733	-1	-5.523859	4.196858	0.018183048
LNCPRESS2	4	92268767	92277075	-1	4.5554	4.478322	0.024258454

AC092490.1	12	8788257	8795789	1	4.972019	6.759198	0.003453123
TMEM158	3	45224466	45226278	-1	-3.707729	5.102853	0.025211153
HMGB1P3	2	230515023	230515638	1	-6.561605	2.980568	0.043831579
PURPL	5	27472292	27496401	1	4.072857	4.146691	0.041078759
TMED7-TICAM2	5	115578642	115626161	-1	-3.859652	3.641016	0.046557785
SNORD116-26	15	25099499	25099594	1	3.45771	5.878346	0.030421178
RN7SKP11	12	96427200	96427486	-1	-6.642335	3.041763	0.043831579
Y_RNA	11	47820200	47820289	-1	7.079471	3.386397	0.022250379
AC104257.1	8	131308545	131317632	1	4.685753	4.208929	0.018183048
AC009446.1	8	71675300	71702786	1	5.058894	3.150677	0.037646741
AC064802.1	8	114282067	114295839	1	5.105392	6.205045	0.004151863
NPIPA2	16	14748066	14765413	1	4.388846	3.893125	0.032594146
LINC00678	11	27617626	27634627	-1	5.246418	5.966474	0.00392419
TBX5-AS1	12	114408191	114412831	1	-6.842537	3.196992	0.032634429
AC069208.1	12	24213256	24562590	-1	-3.94829	3.77426	0.038694787
AL355102.2	14	96204844	96263929	1	-7.065122	3.853125	0.010068124
HOXB7	17	48607227	48633572	-1	-10.53172	6.570977	0.000112986
LINC01896	18	78976555	78979074	-1	4.828733	3.398125	0.025109373
MIR3687-2	21	8987370	8987430	1	8.223794	4.375386	0.004343433
AC016596.1	5	55944656	55944733	1	10.83406	6.86768	6.95E-05
ESRG	3	54632122	54639857	-1	5.096729	5.386409	0.00571431
AC011447.3	19	20125377	20321305	-1	3.698877	6.455051	0.021473159
FENDRR	16	86474529	86509099	-1	-9.1111	6.133705	0.000234269
AC011530.1	19	45779437	45785973	-1	-6.518799	4.441198	0.003871538
AC008753.2	19	53788782	53789168	1	6.669404	3.062146	0.037646741
AC242498.1	1	144965025	144965135	1	-6.761936	3.133917	0.037646741
RNU6-6P	10	13217269	13217375	1	10.46067	6.50138	0.000126845
CD24	6	106969831	106975627	-1	5.290406	7.874788	0.001823754
AC010735.2	2	226800146	226811029	1	-5.9533	3.222878	0.032634429
RMST_2	12	97492462	97492597	1	4.18738	4.823857	0.024349562
FAM27E3	9	67717411	67719178	-1	3.530834	4.135952	0.043901897
U1	1	146409903	146410062	1	6.603235	3.011695	0.043831579
ZNFX1-AS1_2	20	49279116	49279208	1	-7.211233	3.494764	0.019833567
SNORD50B	6	85677589	85677658	-1	8.189391	4.344249	0.004610543
RNA5-8S5	KI270733.1	173956	174108	1	-4.551154	4.084618	0.022261254
MIAT_exon5_2	22	26672164	26672248	1	8.283576	4.429658	0.003871538
SNORD27	11	62855012	62855083	-1	-3.666313	4.984075	0.0301165
TUG1_2	22	30971296	30971382	1	-6.934043	3.269513	0.028521587
RMST_9	12	97560856	97561048	1	7.49888	3.736314	0.013150652
JPX_2	X	73944595	73944663	1	-7.075014	3.383057	0.022250379
SNORA9	7	44985378	44985510	-1	3.972414	4.590793	0.036401358
MIR3687-1	21	8208844	8208904	1	8.223794	4.375386	0.004343433
U1	1	144560666	144560829	-1	-4.422153	5.190663	0.01363195
ZNFX1-AS1_3	20	49280485	49280571	1	8.500638	4.628378	0.002826005

PWAR5	15	24985053	24988232	1	4.226501	4.949314	0.019995419
SNORA16A	1	28580920	28581054	-1	7.561358	3.789821	0.011989555
AC243547.3	1	145927258	145977811	1	-3.891514	4.522769	0.040207296
AC025048.6	17	60101759	60102919	-1	-3.939218	5.063668	0.026096257
AL591742.2	1	114032393	114034511	1	5.475293	4.61002	0.009297076

Table S6. GO enrichment analysis results

Category	Term	Count	%	P-Value	Benjamini
GOTERM_BP_DIRECT	extracellular matrix organization	29	6.4	1.00E-15	2.20E-12
GOTERM_BP_DIRECT	cell adhesion	33	7.3	3.00E-09	3.30E-06
GOTERM_BP_DIRECT	response to drug	26	5.8	6.70E-09	4.90E-06
GOTERM_BP_DIRECT	collagen catabolic process	13	2.9	7.60E-09	4.20E-06
GOTERM_CC_DIRECT	extracellular matrix	36	8	4.50E-17	1.50E-14
GOTERM_CC_DIRECT	extracellular region	85	18.8	6.80E-16	1.10E-13
GOTERM_CC_DIRECT	extracellular space	75	16.6	3.70E-15	4.20E-13
GOTERM_CC_DIRECT	focal adhesion	35	7.7	1.30E-12	1.10E-10
GOTERM_CC_DIRECT	proteinaceous extracellular matrix	28	6.2	1.00E-11	6.90E-10
GOTERM_CC_DIRECT	extracellular exosome	106	23.5	2.20E-10	1.20E-08
